# Defensive Responses of Tea Plants (*Camellia sinensis*) Against Tea Green Leafhopper Attack: A Multi-Omics Study

**DOI:** 10.3389/fpls.2019.01705

**Published:** 2020-01-17

**Authors:** Xiaoman Zhao, Si Chen, Shanshan Wang, Wenna Shan, Xiaxia Wang, Yuzhen Lin, Feng Su, Zhenbiao Yang, Xiaomin Yu

**Affiliations:** ^1^College of Horticulture, Fujian Agriculture and Forestry University, Fuzhou, China; ^2^FAFU-UCR Joint Center for Horticultural Biology and Metabolomics, Fujian Provincial Key Laboratory of Haixia Applied Plant Systems Biology, Fujian Agriculture and Forestry University, Fuzhou, China; ^3^Fujian Farming Technology Extension Center, Fuzhou, China; ^4^Center for Plant Cell Biology, Department of Botany and Plant Sciences, Institute for Integrative Genome Biology, University of California, Riverside, Riverside, CA, United States

**Keywords:** plant-herbivore interaction, tea green leafhopper, defense response, hormone signaling, metabolomics, RNA-Seq

## Abstract

Tea green leafhopper [*Empoasca* (*Matsumurasca*) *onukii* Matsuda] is one of the most devastating pests of tea plants (*Camellia sinensis*), greatly impacting tea yield and quality. A thorough understanding of the interactions between the tea green leafhopper and the tea plant would facilitate a better pest management. To gain more insights into the molecular and biochemical mechanisms behind their interactions, a combined analysis of the global transcriptome and metabolome reconfiguration of the tea plant challenged with tea green leafhoppers was performed for the first time, complemented with phytohormone analysis. Non-targeted metabolomics analysis by ultra-performance liquid chromatography quadrupole time-of-flight mass spectrometry (UPLC-QTOF MS), together with quantifications by ultra-performance liquid chromatography triple quadrupole mass spectrometry (UPLC-QqQ MS), revealed a marked accumulation of various flavonoid compounds and glycosidically bound volatiles but a great reduction in the level of amino acids and glutathione upon leaf herbivory. RNA-Seq data analysis showed a clear modulation of processes related to plant defense. Genes pertaining to the biosynthesis of phenylpropanoids and flavonoids, plant-pathogen interactions, and the biosynthesis of cuticle wax were significantly up-regulated. In particular, the transcript level for a *CER1* homolog involved in cuticular wax alkane formation was most drastically elevated and an increase in C29 alkane levels in tea leaf waxes was observed. The tea green leafhopper attack triggered a significant increase in salicylic acid (SA) and a minor increase in jasmonic acid (JA) in infested tea leaves. Moreover, transcription factors (TFs) constitute a large portion of differentially expressed genes, with several TFs families likely involved in SA and JA signaling being significantly induced by tea green leafhopper feeding. This study presents a valuable resource for uncovering insect-induced genes and metabolites, which can potentially be used to enhance insect resistance in tea plants.

## Introduction

Plants as sessile organisms are constantly attacked by a wide range of herbivorous insects. It has been estimated that foliage, sap feeding, and root herbivory cause more than 20% reduction in annual net plant productivity (Agrawal, 2011). To cope with herbivore challenges, plants have developed a battery of sophisticated mechanisms to fence against herbivore attacks, while maintaining functional flexibility and minimizing impacts on plant fitness (Baldwin and Preston, 1999; Yang et al., 2015). Plants confront herbivores by launching direct or indirect defenses. Physical barriers like hairs, trichomes, thorns, waxy cuticles, and spines are typical examples of direct defenses (War et al., 2012). In face of phytophagous threats, plants also produce a plethora of specialized metabolites such as glucosinolates, alkaloids, phenolics, and terpenoids, acting as repellents, antidigestives, or toxins (Wu and Baldwin, 2010). Indirect defenses do not directly impact insect herbivores but can attract their natural enemies by emitting a blend of VOCs called herbivore-induced plant volatiles (HIPVs) so as to prevent further damage to plant tissues (Aljbory and Chen, 2018). Plant defenses can also be classified as being constitutive or inducible and the latter allows energy allocation to growth and reproduction in the absence of insect attack, thus reducing the metabolic costs in plants (Zhou et al., 2015).

Upon the perception of feeders, plants trigger a cascade of phytohormone-modulated signal transduction pathways to mount specific phenotypic responses according to the nature and the duration of damage. The plant defense responses to insects also vary depending on plant species, ecotypes, and developmental stages (Santamaria et al., 2018). It is generally believed that phytohormones jasmonic acid (JA), salicylic acid (SA), and ethylene (ET) are major regulators of plant defense responses. In addition, auxin, abscisic acid (ABA), gibberellin (GA), brassinosteroid (BR), and cytokinin (CK) may also be involved in plant interactions with pathogens and insect pests (Yang et al., 2015). Generally, plant responses to chewing insects (*e.g.*, caterpillars and beetles) that consume large portions of plant tissues differ from those to piercing-sucking insects (*e.g.*, aphids), which feed on the vascular systems of plants with their stylets and cause minimal tissue disruption. The classical view is that JA is primarily involved in activating the response against herbivorous insects and necrotrophic pathogens while SA is responsible for defense against biotrophic and hemi-biotrophic pathogens (Bari and Jones, 2009). Accumulating evidence has shown that the crosstalk among various hormones to coordinate the expression of genes and their regulators is key to eliciting efficient stress response (Verma et al., 2016).

Tea is one of the most consumed non-alcoholic beverages in the world, owing its popularity to delight flavors and a multitude of health-promoting benefits (Khan and Mukhtar, 2013). Like other plants, the tea plant (*Camellia sinensis*) suffers heavily from herbivore pressure during its life cycles. Approximately 1,034 arthropod species are associated with tea, with 3% being prevalently found throughout the world (Chen and Chen, 1989). These insect herbivores impose a severe constraint to tea production, leading to 11%–55% yield loss globally (Hazarika et al., 2009). The tea green leafhopper, *Empoasca* (*Matsumurasca*) *onukii* Matsuda, is a polyphagous phloem-feeding specialist pest of tea plants in Asian tea growing regions (Qin et al., 2015). There are usually 10–17 generations per year. Nymphs and adults suck phloem sap of tender shoots, buds and leaves and the female adults lay eggs within the stem of tender shoots. Leaf curling and parching as well as vein reddening are early damage symptoms caused by tea green leafhoppers. Continuous feeding results in defoliation and halts plant growth, thereby leading to severe yield loss and dramatic decrease in tea quality (Jin et al., 2012). *E*. (*M*.) *onukii* has become one of the most serious tea plant insects in East Asia. Currently, the control of this insect relies heavily on the use of chemical pesticides, which raises the potential problems of environmental pollution and the development of insecticide resistance (Wei et al., 2017).

Studies on the interaction between tea plants and tea green leafhoppers have largely focused on volatile compounds. Tea green leafhopper feeding not only enhances the expression of terpene synthase genes and hence the emission of (*S*)-linalool and geraniol, but also induces diendiol I production, a volatile monoterpene thought to be a unique VOC marker of *E*. (*M*.) *onukii*-infested tea leaves (Mei et al., 2017; Zeng et al., 2018). Given that the mild-infestation by tea green leafhoppers can make tea more aromatic, in practical tea applications, tea workers have taken advantage of this interaction to make “Oriental Beauty”, a famous Taiwan oolong tea (Cho et al., 2007). Some progresses have also been made towards understanding the mechanisms for resistance against tea green leafhoppers. A total of 21 genes obtained from a subtractive cDNA library of a tea plant were found to be up-regulated by mild-infestation of tea green leafhoppers and these genes are mainly involved in stress response, specialized metabolism, and carbohydrate metabolic processes (Yang et al., 2011). In a very recent study, targeted analyses of tea green leafhopper and tea geometrid feeding on levels of tea metabolites (catechins, caffeine and theanine) and phytohormones were performed, showing that both insect attacks did not significantly alter the contents of catechins and caffeine. The theanine level was increased by tea geometrid feeding but was not significantly affected by tea green leafhopper feeding. In addition, both insect attacks increased the contents of JA and SA (Liao et al., 2019). Nonetheless, the aforementioned experiment was performed solely on plucked tea leaves, whose reactions to insect attacks would assumingly differ from those of a living plant. How does a living tea plant react to the insect damage? Beyond catechins, caffeine, and theanine, to what extent is the tea plant metabolism reprogrammed upon leaf herbivory? What is the molecular mechanism behind the induced defense responses to the tea green leafhopper attack? To answer these questions, here, we performed for the first time the integrated analysis of the global transcriptome and metabolome profiles of tea plants challenged with *E*. (*M*.) *onukii*. By this multi-omics approach, we identified specialized metabolites, genes, and metabolic pathways likely important for conferring insect resistance. Furthermore, we determined the effects of tea green leafhopper feeding on leaf wax compositions and phytohormone levels in tea plants. Lastly, we discussed the hormone signaling and transcription factor families potentially related to modulating defense responses. The results obtained herein would provide a useful resource of defense genes and metabolites from tea plants that would be beneficial for the development of sustainable and environmentally safe pest control strategies.

## Materials and Methods

### Plant Materials and Treatments

Clonal cuttings of 2-year-old tea plants (*C. sinensis* cv. “Jinxuan”) with one seedling per plastic pot (25 cm diameter × 30 cm height) were used in the current study. Plants were kept in the greenhouse of Fujian Agriculture and Forestry University (FAFU), Fuzhou, China with light and dark cycles of 12 and 12 h at 25 ± 3°C with 65 ± 5% RH. All of the potted plants were watered and fertilized by the same standards for one year. Tea plants showing uniform growth and no signs of disease or insect damage were chosen for our experiments.

*E*. (*M*.) *onukii* Matsuda adults and nymphs of mixed age and sex were collected using a sweep net from tea plants at the Tea Plantation of FAFU. They were reared on fresh tea leaves of “Jinxuan” tea cultivar in a ventilated nylon cage (80 cm × 80 cm × 60 cm) housed in the climate-controlled greenhouse as described above. To constrain insects, 16 potted plants subjected to insect treatment were covered with a nylon cage (100 cm × 100 cm × 100 cm). For insect infestation, these caged plants were exposed to approximately 100 tea green leafhoppers that were replenished every two days for up to 3 weeks to obtain leafhopper-damaged tea samples (LD). For the mechanical damage treatment, four to five shoots comprising one bud and two leaves from each of the other five potted plants were pricked evenly in the whole bud and leaf area using a microcapillary needle with six pricks per time. Pricking was repeated every 2 days for the same duration as the insect treatment to obtain mechanically-damaged tea samples (MD). The microcapillary needle was 50 µm in diameter so as to simulate the size of the maxillary stylets of *Empoasca* (Tavella and Arzone, 1993). The remaining five undamaged potted plants served as the control (CK). To prevent the interference of other insects, tea plants in MD and CK groups were also covered with nylon cages.

Treatment time was established according to the intensity of herbivore damage on tea leaves. After 3 weeks of treatment, early symptoms of tea green leafhopper damage such as leaf curling, chlorosis, and some parching on leaf edge began to develop on infested tea leaves (**Supplemental Figure S1** in **Data Sheet 1**). Damaged tea shoots comprising one bud and two leaves were then plucked from the LD and MD groups. In the meantime, tea shoots comprising one bud and two leaves were plucked from the CK group. Nine biological replicates were harvested from each group, with three each for RNA-Seq, metabolomic analysis, and hormone measurements, respectively. These samples were quickly frozen in liquid nitrogen and stored at -80°C for further use. Another three biological replicates were harvested from the LD and CK groups. The first and the second leaves were quickly dissected, combined, and used for the analysis of leaf wax compositions.

### UPLC-QTOF MS Based Metabolomic Analysis

In total, nine samples (three biological replicates per treatment group) were analyzed by ultra-performance liquid chromatography quadrupole time-of-flight mass spectrometry (UPLC-QTOF MS) based metabolomic approach. Tea samples were extracted with 70% (v/v) methanol following our previously published protocol (Chen et al., 2018). One microliter of the metabolite extract was injected into an Acquity UPLC system coupled to a SYNAPT G2-Si HDMS QTOF mass spectrometer (Waters, Milford, MA, USA) and separated with an Acquity UPLC HSS T3 column (2.1×100 mm, 1.8 µm). The instrument setup was the same as previously described (Chen et al., 2018). Progenesis QI software (ver 2.1, Nonlinear Dynamics, Newcastle upon Tyne, UK) with default settings was used for data preprocessing. The preprocessed dataset was then exported to Progenesis QI extension EZinfo (ver 3.0), pareto-scaled and subjected to multivariate data analysis, such as principal component analysis (PCA) and supervised partial least squared discriminant analysis (PLS-DA). Variable importance in projection (VIP) analysis was performed to evaluate the importance of metabolites for group separation. One-way analysis of variance (ANOVA) was carried out using SPSS (ver 13.0, Chicago, IL, USA) and differences between means were determined by Tukey's HSD (honestly significance difference) test. Differentially expressed metabolites (DEMs) among different treatment groups were selected with VIP> 1 and a *p* value < 0.05. Compound assignments for DEMs were performed using the following methods. For DEMs where authentic standards were available, metabolite identification was verified by running authentic standards in exactly the same UPLC and MS conditions as the biological samples. Then the retention time and MS/MS fragment ions derived from the authentic standard was compared with that from the samples. If the authentic standards were not available, tentative metabolite assignments were made by comparing the mass spectra and UV absorbance (if available) with those from online spectral databases such as Metlin (Tautenhahn et al., 2012), ReSpect (Sawada et al., 2012), MassBank (Horai et al., 2010), and KNApSAcK (Afendi et al., 2012), as well as literature references. Standardized log2 abundance values of the identified DEMs were shown in the heatmap using MultiExperiment Viewer software (MeV ver 4.9.0, J. Craig Venter Institute, La Jolla, CA, USA).

### Quantifications of Catechins, Caffeine and Amino Acids

To detect catechins, theanine, and caffeine, two microliters of 500-fold diluted tea extracts were injected on an Acquity UPLC system coupled to a XEVO TQ-S MS triple quadrupole mass spectrometer (Waters, Milford, MA, USA) and separated in an Acquity UPLC BEH C18 column (2.1 × 100 mm, 1.7 µm). To detect amino acids, 25-fold diluted tea extracts was separated on a Merck SeQuant ZIC-HILIC column (2.1 × 100 mm, 5 µm). Chromatographic conditions and the equipment setup were described in our previous study (Chen et al., 2018). Contents of catechins, caffeine and amino acids in tea samples were calculated by comparison of their peak areas with those of standards in the calibration curve.

### Phytohormone Analysis

Phytohormone extractions were performed by using a previously reported method (Yang et al., 2019) with some modifications. Briefly, after the addition of internal standards including 50 ng of (±)-[^2^H_6_]JA, 10 ng of [^2^H_4_]SA, and 10 ng of [^2^H_6_]ABA, 100 mg (fresh weight) of finely powdered tea samples was extracted with 1 mL of ethyl acetate. Samples were vortexed for 10 min and centrifuged at 14,000 *g* for 15 min at 4°C. The resulting supernatant was transferred and vacuum evaporated until total dryness at room temperature. The dried residues were redissolved in 500 μl of 70% methanol and filtered through a 0.22 µm PVDF filter (Millipore). Samples were analyzed *via* UPLC-XEVO TQ-S MS triple quadrupole mass spectrometer (Waters, Milford, MA, USA) equipped with an Acquity UPLC BEH C18 column (2.1 × 100 mm, 1.7 µm) thermostatted at 40°C. Separation was performed with water containing 5 mM ammonium formate (phase A) and methanol (phase B) as mobile phases. The chromatographic gradient was as follows: 0–13 min (95%–0% A), 13–17 (0%–0% A), and 17–17.1 min (0%–95% A). The flow rate was set at 0.3 ml/min. The mass spectrometer was operated in ESI^-^ mode with the following settings: capillary voltage: 1.27 kV; desolvation temperature: 400°C; source temperature: 150°C; cone gas flow: 150 L/h; desolvation gas flow: 800 L/h. Collision energy and cone voltage for measured hormones were individually optimized with MRM for quantification using authentic standards. The precursor and product ions for JA were 209.3 and 59.3, respectively. The precursor and product ions for SA were 137.1 and 65.2, respectively. The precursor and product ions for ABA were 263.2 and 153.2, respectively. JA, SA, and ABA contents were quantified based on the ratio between their ion intensities and the respective internal standards.

### Leaf Cuticular Wax Analysis

To compare the differences in leaf wax compositions between CK and LD groups, total cuticular wax was extracted and analyzed as previously described (Zhu et al., 2018). Tea leaves were soaked with 5 ml of chloroform containing 10 µg of *n*-tetracosane (internal standard) for 30 s at room temperature. The liquid was transferred and vacuum-dried to complete dryness. Samples were derivatized at 70°C for 1 h in 50 µl of *N*,*O*-bis(trimethylsilyl)trifluoroacetamide (BSTFA, Sigma-Aldrich) containing 1% trimethylchlorosilane (Sigma-Aldrich) and 200 µl of anhydrous pyridine (Sigma-Aldrich, 99.8% purity). The liquid was vacuum evaporated to complete dryness again and redissolved in 150 µl of chloroform. Aliquots of derivatized samples were separately injected into GCMS-QP2010 Ultra (Shimadzu, Japan) and GC-2010 Plus (Shimadzu, Japan) systems both equipped with an Agilent DB-1 capillary column (30 m x 0.25 mm i.d., 0.25 µm film thickness) for gas chromatography-mass spectrometry (GC-MS) and gas chromatography-flame ionization detector (GC-FID) analyses, respectively. The instrument settings were exactly the same as previously reported (Zhu et al., 2018). The alkane mix standard comprising C10 to C40 was diluted to 100 ng/µl with hexane and 1 µl was injected into GC-MS and GC-FID to assist with metabolite identification. The MS detector was used for compound identification by comparing the mass spectra with the alkane mix standards or with references while the FID detector was used for compound quantification after normalization to the peak area of the internal standard. The total amount of leaf wax was expressed per unit of leaf surface area, which was measured by ImageJ software (http://rsb.info.nih.gov/ij/) based on the apparent leaf blade areas in digital images.

### RNA Sequencing, Annotation, and Expression Analysis

A total of nine samples (three biological replicates per treatment group) were used for RNA extraction. Total RNAs were extracted with the RNAprep Pure Plant Kit (Tiangen, Beijing, China) following the manufacturer's procedures. RNA integrity was assessed using an Agilent Bioanalyzer 2100 (Agilent Technologies, CA, USA). Sequencing libraries were generated using the TruSeq Stranded Total RNA Library Prep Kit according to the manufacturer's instructions (Illumina, USA). Libraries were quantified on an Agilent Bioanalyzer 2100 (Agilent Technologies, CA, USA). The cDNA was shotgun sequenced (150-bp paired-end reads) on an Illumina HiSeq 4000 system using a customer sequencing service (Novogene Bioinformatics Technology Co., Ltd, Beijing, China).

To obtain clean reads, raw reads were processed through in-house perl scripts to filter reads with adapter sequences, ambiguous nucleotides (>5%) or low quality bases (>20% of the bases with a quality score of 10). *C. sinensis* cv. “Suchazao” genome sequences and gene annotation files were downloaded from the tea genome website (http://tpia.teaplant.org/). The index of the reference genome was built and paired-end clean reads were aligned to the reference genome with the Bowtie software (Langmead et al., 2009). Read numbers mapped to each gene of the reference genome were counted using featureCounts (ver 1.5.0). Fragments per kilobase of exon model per million mapped reads (FPKM) were used to quantify transcript abundance. The DESeq2 R package (ver 1.16.1) in R (Anders and Huber, 2010) was applied to identify differentially expressed genes (DEGs), which were defined as genes with *p* < 0.05 and an absolute value of log2 (fold change) > 0.5. Kyoto Encyclopedia of Genes and Genomes (KEGG) and Gene Ontology (GO) enrichment analyses of DEGs were performed by using the clusterProfiler R package.

### qRT-PCR Validation for DEGs

To verify the accuracy of RNA-Seq results, a total of 13 DEGs involved in flavonoid, theanine, terpene, JA, and wax biosynthesis were selected for qRT-PCR analysis. Gene specific primers were designed (**Supplemental Table S1**). The qRT-PCR was performed in a 20 μl reaction volume with 1.0 μl cDNA, 0.4 μM of each primer, and SYBR Premix EX Taq™ II (Takara) on a CFX96™ real-time PCR system (Bio-Rad, USA) under the following parameters: 95°C for 30 s, 40 cycles of 95°C for 5 s and 60°C for 30 s. Reactions were run in triplicates. GAPDH sequence (accession no. KA295375.1) from the tea plant was used as a reference gene. Relative gene expression was calculated using the 2-^ΔΔ^Ct method (Livak and Schmittgen, 2001). No-template controls were included in each run.

### Chemicals and Reagents

Acetonitrile (MS grade) and methanol (MS grade) were obtained from Thermo Fisher Scientific, Inc. (Pittsburgh, PA, USA). Formic acid and ammonium formate were purchased from Honeywell Fluka (Seelze, Germany). Deionized water was produced by a Milli-Q water purification system (Millipore, Billerica, MA, USA). L-theanine, (-)-epigallocatechin gallate (EGCG), (-)-epigallocatechin (EGC), (+)-catechin (C), (-)-epicatechin gallate (ECG), (-)-epicatechin (EC), and (-)-gallocatechin (GC) were obtained from Sigma-Aldrich (St. Louis, MO, USA). Epigallocatechin 3-(3-*O*-methylgallate) (EGCG3″Me) (≥95%) was purchased from ChemFaces (Wuhan, China). Caffeine (≥98%) was obtained from Yuanye Biotechnology Inc. (Shanghai, China). (±)-[^2^H_6_]JA was purchased from CFW Laboratories Inc. (Walnut, CA, USA). [^2^H_4_]SA was purchased from Sigma-Aldrich (St. Louis, MO, USA). [^2^H_6_]ABA was purchased from Olchemlm Ltd. (Olomouc, Czech Republic). An alkane mix standard (C10-C40) with ≥95% purity was purchased from AccuStandard Inc. (New Haven, CT, USA).

### Access Code

RNA-Seq raw data were deposited to NCBI Sequencing Read Archive database and could be accessed under accession number PRJNA553681.

## Results

### Tea Green Leafhopper Infestation Elevates the Production of Defensive Specialized Metabolites in Infested Plants

**Figure 1:**
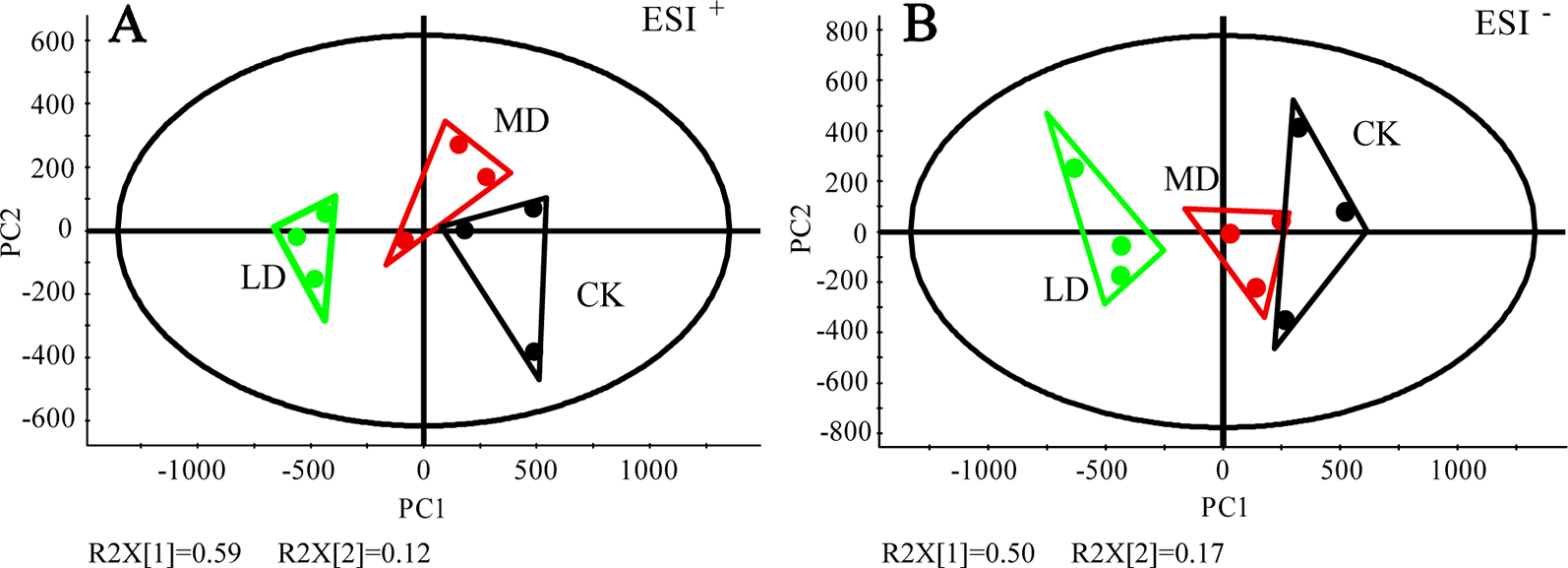
PCA analysis of tea leaves exposed to tea green leafhopper attack and mechanical damage. **(A)** PCA score plot for tea samples based on 2,381 molecular features detected in ESI^+^. **(B)** PCA score plot for tea samples based on 906 molecular features detected in ESI^-^. R2X, explained variation. PC1, the first principal component. PC2, the second principal component. For tea samples with different treatments, three biological replicates were prepared, where one replicate was a pool of collected materials from different tea plants. LD, tea green leafhopper-infested leaves; MD, mechanically damaged leaves; CK, undamaged control.

We investigated the effects of tea green leafhopper feeding on the metabolic response of “Jinxuan”, a widely grown tea cultivar in South China. Tea leaves sampled from mechanically damaged tea plants (MD group) and uninfested tea plants (CK group) were used as controls. To gain an overview of the metabolome changes induced by different treatments, non-targeted metabolomics analysis of tea samples was performed *via* UPLC-QTOF MS in both ESI^+^ and ESI^-^ modes, which detected 2, 381 and 906 mass/retention time features, respectively (**Supplemental Table S2**). As with other tea cultivars, common constituents such as flavanols, proanthocyanidins, flavonol glycosides, and purine alkaloids were abundantly detected in “Jinxuan” tea cultivar (**Supplemental Table S2**). PCA analysis of the resulting metabolite data revealed a clear separation of the tea green leafhopper-damaged group (LD group) from the MD and CK groups in both modes, while the latter two groups were not well separated (**Figures 1A, B**). Score plots from the PLS-DA model showed a distinct separation among treatments (**Supplemental Figure S2** in **Data Sheet 1**). The model parameters were as follows: goodness of fit R^2^Y = 0.883 (ESI^+^) and 0.864 (ESI^-^), predictive ability Q^2^ = 0.569 (ESI^+^) and 0.562 (ESI^-^). By filtering with VIP> 1 and a *p* value <0.05, 205 and 50 molecular features were subsequently found to be differentially accumulated in respective modes (**Supplemental Table S2**). Complemented with the untargeted analysis, the absolute contents of three important metabolite classes in tea plants-catechins, caffeine and amino acids-were measured by UPLC-QqQ MS (**Supplemental Table S3**). After removing duplicated signals detected in both modes and daughter ions derived from collision-induced dissociation, we detected 123 DEMs combining untargeted and targeted analyses, from which 25 DEMs were identified or tentatively identified based on the accurate mass and MS/MS fragmentation patterns compared with authentic standards and literature references (**Table 1**). They were assigned to eight metabolite classes, including amino acids and peptides, hydrolysable tannins, flavanols, flavonol glycosides, flavone glycosides, flavanone glycosides, proanthocyanidins, and glycosidically bound volatiles.

**Table 1:** Tentative assignments of differentially expressed metabolites in tea plants in response to tea green leafhopper attack and mechanical damage by UPLC-QTOF MS and UPLC-QqQ MS.

Tentative assignments	Metabolite class	RT (min)	Formula	Detected [M − H]^−^ (*m*/*z*)	Theoretical [M − H]^−^ (*m*/*z*)	ΔMass (ppm)	MS/MS fragments	Reference
L-theanine	Amino acids and peptides	1.40	C_7_H_14_N_2_O_3_	173.0926	173.0926	0.00	155.0818, 128.0350	Authentic standard^b^
Glutathione	Amino acids and peptides	1.53	C_10_H_17_N_3_O_6_S	306.0757	306.0760	-0.98	272.0881, 254.0775, 230.9514, 210.0878	Authentic standard^b^
Methyl 6-*O*-galloyl *β* -D-glucose	Hydrolysable tannins	3.65	C_14_H_18_O_10_	345.0819	345.0822	-0.87	225.0394, 183.0288	(Tautenhahn et al., 2012)
EGC	Flavanols	4.91	C_15_H_14_O_7_	305.0666	305.0661	-1.64	219.0655, 179.0348, 165.0191, 137.0243, 125.0242	Authentic standard^b^
Benzyl *β*-primeveroside	Glycosidically bound volatiles	6.02	C_18_H_26_O_10_	403.1585^a^	403.1604^a^	-4.71	245.0448	(Miyase et al., 1988)
EGC-ECG dimer	Proanthocyanidins	6.04	C_37_H_30_O_17_	745.1409	745.1405	0.54	611.1611, 407.0757, 289.0708, 149.0238	(Jiang et al., 2015)
Isovitexin glucoside	Flavone glycosides	6.05	C_27_H_30_O_15_	595.1649^a^	595.1663^a^	-2.36	433.1119, 313.0699	(Ferreres et al., 2003)
Carthamidin 6,7-diglucoside	Flavanone glycosides	6.05	C_27_H_32_O_16._	611.1615	611.1612	0.49	329.0963, 149.0292	(Wishart et al., 2018)
Naringenin diglucoside	Flavanone glycosides	6.13	C_27_H_32_O_15_	595.1660	595.1663	-0.50	475.1215	(Tautenhahn et al., 2012)
EC	Flavanols	6.25	C_15_H_14_O_6_	289.0713	289.0712	0.35	245.0813, 203.0704, 137.0238, 123.0445	Authentic standard^b^
EGCG	Flavanols	6.34	C_22_H_18_O_11_	457.0776	457.0771	1.09	305.0666, 169.0142, 125.0240	Authentic standard^b^
EC-ECG dimer	Proanthocyanidins	6.45	C_37_H_30_O_16_	731.1590^a^	731.1612^a^	-3.00	407.0726,393.0976,195.0872	(Nonaka et al., 1983)
Prodelphinidin A2 3**'**-gallate	Proanthocyanidins	7.00	C_37_H_28_O_18_	759.1224	759.1197	3.56	607.1103,589.0945	(Wishart et al., 2018)
Kaempferol deoxyhexose-hexose-deoxyhexose	Flavonol glycosides	7.34	C_33_H_40_O_19_	739.2071	739.2086	-2.03	(ESI^+^) 595.1650, 433.1123, 287.0545	(Sawada et al., 2009)
EGCG3**''**Me	Flavanols	7.42	C_23_H_20_O_11_	471.0929	471.0927	0.42	305.0663, 287.0554,183.0298,161.0238	Authentic standard^b^
Camellianin B	Flavone glycoside	7.68	C_27_H_30_O_14_	577.1557	577.1557	0.00	413.0854, 293.0438	(Zheng et al., 2015)
L-glutamate**^c^**	Amino acids and peptides	7.73	C_5_H_9_NO_4_	148.20^a^	148.06^a^	/	102.2, 84.2	Authentic standard^b^
ECG	Flavanols	7.84	C_22_H_18_O_10_	441.0830	441.0822	1.81	383.0071, 289.0717, 245.0810, 169.0139, 125.0241	Authentic standard^b^
Linalool oxide primeveroside isomer 1	Glycosidically bound volatiles	7.90	C_21_H_36_O_11_	465.2334^a^	465.2336^a^	-0.43	355.1716,	(Wishart et al., 2018)
Kaempferol 3-*O*-glucosylrutinoside	Flavonol glycosides	8.00	C_33_H_40_O_20_	755.2037	755.2035	0.26	489.1022, 337.0912, 285.0389, 173.0446	(Dou et al., 2007)
Tricetin	Flavones	8.02	C_15_H_10_O_7_	303.0492^a^	303.0505^a^	-4.29	257.0453,137.0593	(Tautenhahn et al., 2012)
Linalool oxide primeveroside isomer 2	Glycosidically bound volatiles	8.52	C_21_H_36_O_11_	465.2323^a^	465.2336^a^	-2.79	355.1715,335.0952,287.0545	(Wishart et al., 2018)
ECG3**''**Me	Flavanols	8.89	C_23_H_20_O_10_	455.0981	455.0978	0.66	289.0717, 183.0297	Authentic standard^b^
Epiafzelechin 3-gallate	Flavanols	8.94	C_22_H_18_O_9_	425.0871	425.0873	-0.47	273.0759, 255.0633	(Tautenhahn et al., 2012)
Linalool primeveroside	Glycodically bound volatiles	11.47	C_21_H_36_O_10_	447.2232	447.2230	0.45	493.2293 ([M+FA-H]^-^), 421.1704	(Tautenhahn et al., 2012)

a [M+H]^+^.

b This letter indicates that identification of the compound was confirmed by the authentic standard.

c This compound was measured on a Merck SeQuant ZIC-HILIC column (2.1 × 100 mm, 5 µm) via UPLC-QqQ MS.

**Figure 2:**
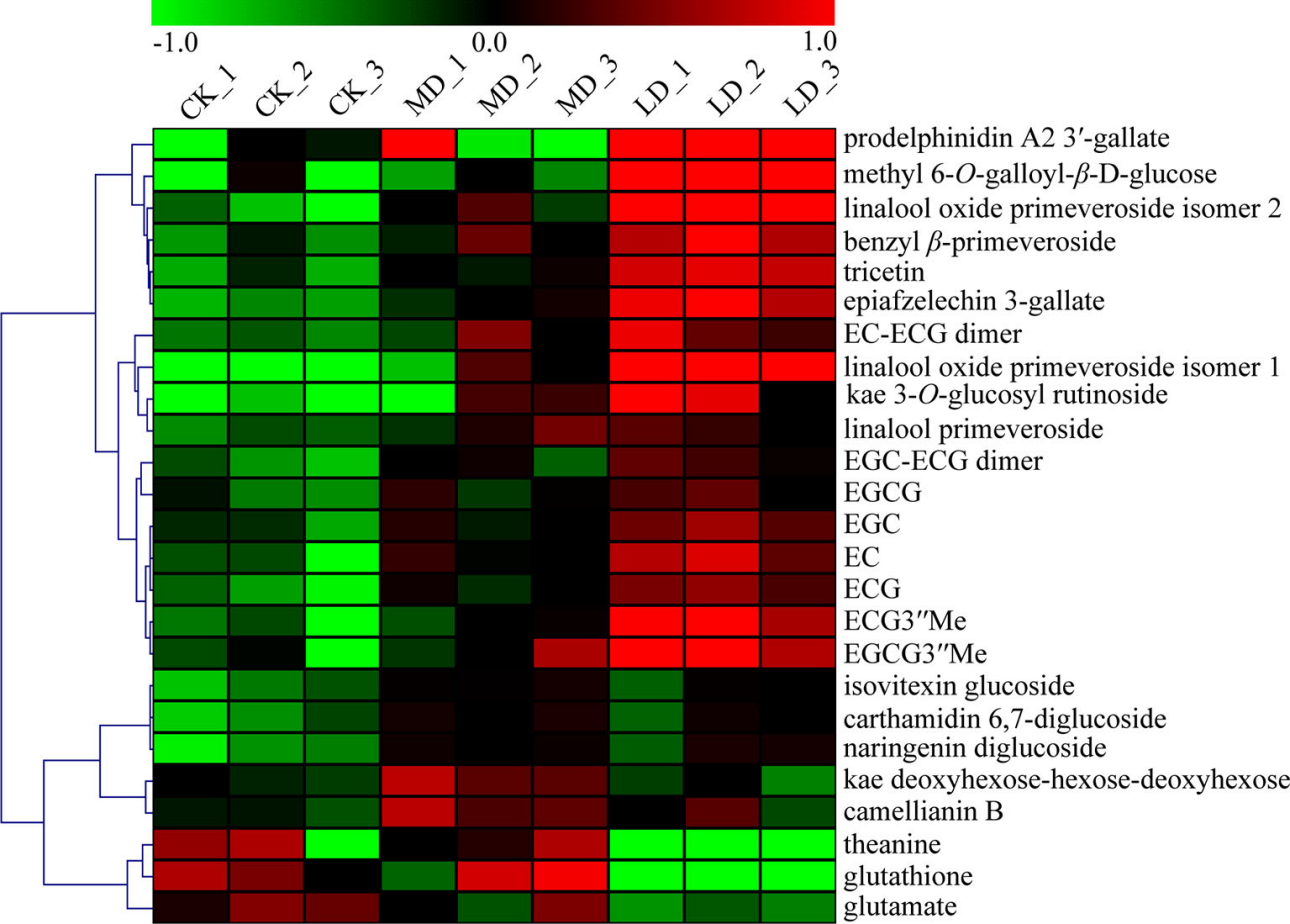
Comparisons of the relative abundance of the identified DEMs among different treatment groups. LD, tea green leafhopper-infested leaves. Kae, kaempferol; MD, mechanically damaged leaves; CK, undamaged control.

A heat map was used to visualize the identified DEMs, showing that tea green leafhopper feeding apparently increased the production of metabolites in the phenylpropanoid and flavonoid pathways in tea leaves (**Figure 2**). For example, major flavanols (EGCG, EGC, ECG, EC, EGCG3”Me, ECG3”Me, and epiafzelechin 3-gallate) showed a clear increase in the LD group. Similar pattern was observed in the accumulation of three proanthocyanidins (EGC-ECG dimer, EC-ECG dimer, and prodelphinidin A2 3′-gallate), one flavone (tricetin), one flavonol glycoside (kaempferol 3-*O*-glucosylrutinoside), and one hydrolysable tannin (methyl 6-*O*-galloyl-*β*-D-glucose). In addition to the variations in flavonoid compositions, four glycosidically bound volatiles including two linalool oxide primeveroside isomers, benzyl primeveroside, and linalool primeveroside were also induced by leaf herbivory. Conversely, leaf herbivory drastically lowered the production of glutathione to almost below the detection limit as well as leading to a 59% reduction in the total amino acid content. Specifically, the levels of predominant amino acids in tea leaves such as theanine, glutamate, aspartate, and serine all declined (**Figure 2**; **Supplemental Table S3**). Caffeine presented no dramatic increase in response to herbivore attack or mechanical damage (**Supplemental Table S3**). For the MD group, except for five flavonoid compounds (camellianin B, isovitexin glucoside, carthamidin 6,7-diglucoside, kaempferol deoxyhexose-hexose-deoxyhexose, and naringenin diglucoside) that accumulated in the highest level, concentrations of other DEMs generally varied between CK and LD groups.

### SA and JA Levels Increase in Tea Green Leafhopper-Infested Plants

**Figure 3:**
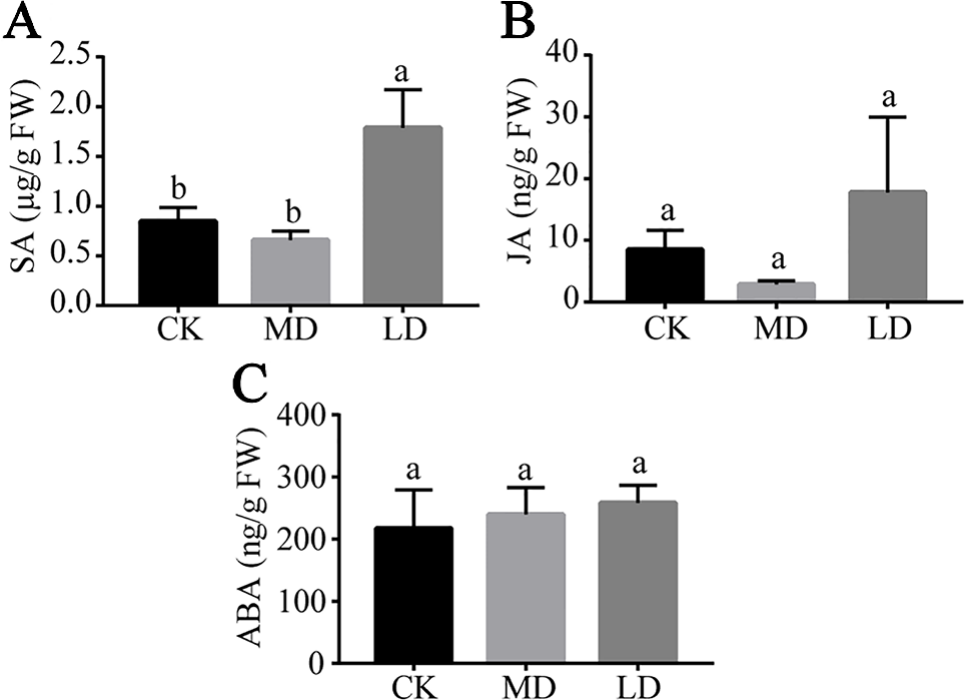
Quantitative analysis of the changes of SA **(A)**, JA **(B)** and ABA **(C)** contents in tea leaves from different treatment groups. Results are expressed as mean ± standard deviation (n = 3). Different letters indicate significant difference (*p* < 0.05) according to Tukey's HSD test. LD, tea green leafhopper-infested leaves; MD, mechanically damaged leaves; CK, undamaged control.

Most plant defense responses against insect herbivores are activated by signal transduction pathways mediated by JA and SA (War et al., 2012). ABA is also an important modulator of the plant immune signaling network (Pieterse et al., 2012). We next investigated the effects of tea green leafhopper attack on the variations of JA, SA, and ABA in tea plants by UPLC-QqQ MS. The most affected phytohormone by leaf herbivory was SA, whose level was prominently elevated in LD samples compared to those in MD and CK (**Figure 3A**). The JA level in LD exhibited an increasing trend compared with controls, albeit not statistically significant (**Figure 3B**). ABA levels stayed relatively stable among different treatment groups (**Figure 3C**).

### Tea Green Leafhoppers Elicit a Drastic Transcriptomic Reprogramming in Infested Plants

To further elucidate the defensive mechanisms of tea plants against tea green leafhopper infestation at the molecular level, we conducted comparative transcriptomics analyses on the aforementioned samples to assess the global response of tea plants induced by this insect. Overall, ~480 million paired-end reads were obtained by Illumina sequencing, with an average of 53.3 million reads per library. All sequenced libraries contained >90% of bases with a quality score >30. Approximately 89.33% of the reads were mapped against the *C. sinensis* cv. “Suchazao” genome, with on average 83.96% of uniquely mapped reads (**Supplemental Table S4**).

**Figure 4:**
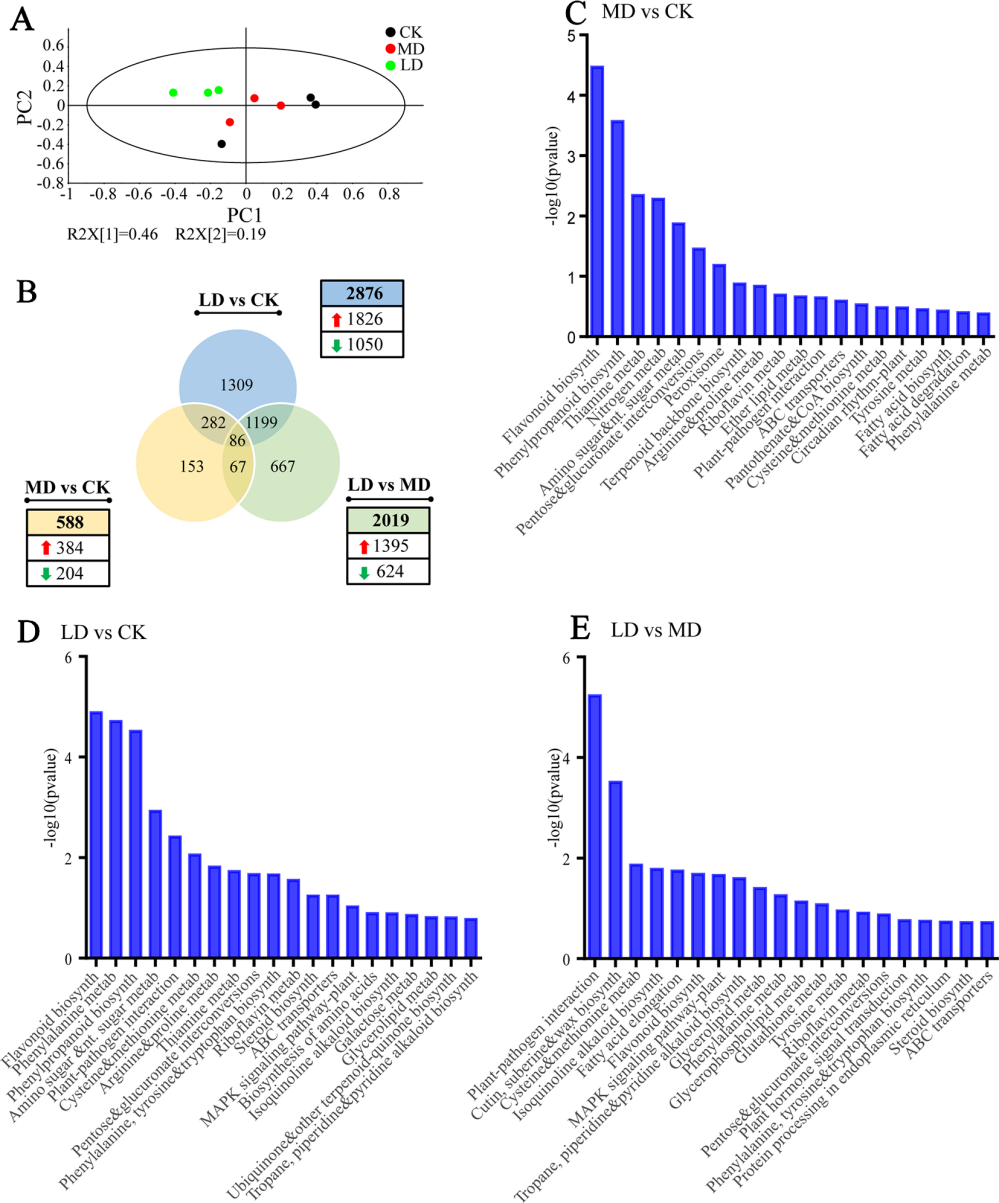
Overview of tea leave transcriptome subjected to mechanical damage and tea green leafhopper infestation. **(A)** PCA score plot based on normalized gene count data from all samples. **(B)** Venn diagram showing the numbers of common and specific DEGs among different treatment groups. **(C)** Analysis of KEGG Orthology (KO) pathway enrichment of DEGs between MD and CK. **(D)** Analysis of KO pathway enrichment of DEGs between LD and CK. **(E)** Analysis of KO pathway enrichment of DEGs between LD and MD. The x axis in **(C)**, **(D)**, and **(E)** represents KEGG pathways while the y axis represents the -log10 (p-value). LD, tea green leafhopper-infested leaves; MD, mechanically damaged leaves; CK, undamaged control.

PCA analysis using normalized gene count data showed that the LD group clustered separately from MD and CK groups while the latter two groups were not well separated, with the first principal component and the second principal component explaining 46% and 19% of the total variance, respectively (**Figure 4A**). This result was in accord with PCA analysis using metabolite data, revealing drastic reprogramming of gene expression induced by leaf herbivory. A comparison of DEGs (*p* < 0.05 and |log_2_ (fold change)| > 0.5) among different groups, as illustrated in the Venn diagram, reflected both common and specific changes in gene expression triggered by different stresses (**Figure 4B**). In comparison to the undamaged control, 2,876 (1,826 up-regulated and 1,050 down-regulated) and 588 (384 up-regulated and 204 down-regulated) genes were found to be differentially expressed in LD and MD groups. Compared with the MD group, the LD group contained 2,019 DEGs, of which 1,395 were up-regulated and 624 were down-regulated. Among three pairwise comparisons, only 86 genes were commonly regulated, whereas 45.5% (1,309/2,876) and 26.0% (153/588) of DEGs were exclusively modulated by herbivore attack and mechanical damage, respectively. Overall, it suggested that tea green leafhopper feeding not only induced more intense changes in transcript levels than the mechanical damage, but also led to more up-regulated rather than down-regulated genes. Thirteen DEGs involved in the biosynthesis of flavonoids, theanine, terpenes, JA, and wax were selected for qRT-PCR. It was shown that the expression patterns of all tested genes were consistent with those obtained by RNA-Seq, indicating that the RNA-Seq results were reliable (**Supplemental Figure S3** in **Data Sheet 1**).

### Tea Green Leafhopper Infestation Induces Defensive Responses in Infested Plants

To classify putative functions of DEGs, KEGG, and GO enrichment analyses were performed to explore overrepresented pathways and biological functions in tea leaves affected by wounding and insect feeding. When comparing MD with CK, we identified the most enriched pathways as “flavonoid biosynthesis”, “phenylpropanoid biosynthesis”, “thiamine metabolism”, “nitrogen metabolism”, “amino sugar and nucleotide sugar metabolism”, and “pentose and glucuronate interconversions” (**Figure 4C**). Differential GO analysis revealed that genes involved in carbon and nitrogen metabolism, transport of sulfur compounds and ions as well as response to oxidative stress were up-regulated whereas genes involved in cellular catabolic process were down-regulated (**Supplemental Tables S5** and **S6**).

In “LD vs CK” comparison, the overrepresented pathways associated with feeding-induced defense included “flavonoid biosynthesis”, “phenylalanine metabolism”, “phenylpropanoid biosynthesis”, “plant-pathogen interaction”, and “phenylalanine, tyrosine, tryptophan biosynthesis” (**Figure 4D**). In addition, the mitogen-activated protein kinase (MAPK) signaling pathway was fairly active in insect-infested tea leaves. Pathways of “phenylalanine metabolism”, “phenylpropanoid biosynthesis”, “flavonoid biosynthesis”, and “phenylalanine, tyrosine and tryptophan biosynthesis” are closely related to the biosynthesis of catechins, proanthocyanidins, flavones, and flavonols. Therefore, the elevated production of flavonoid compounds upon insect attack was most likely the result of induced gene expression in these pathways. GO analysis showed that up-regulated DEGs were mainly linked to carbon and nitrogen metabolism, biosynthesis of aromatic amino acids, lipids, steroids and sulfur compounds, protein modifications and transport of amino acids, organic acids, and anions (**Supplemental Table S7**). On the other hand, the enriched down-represented GO categories contained genes mainly related to photosynthesis, cellular homeostasis, dicarboxylic acid metabolic process, RNA polyadenylation, amine biosynthetic process, and small molecule biosynthetic process (**Supplemental Table S8**).

A further comparison between LD and MD revealed that over-represented KEGG Orthology (KO) terms in LD were mainly related to plant-pathogen interaction, cutin, suberine, and wax biosynthesis, flavonoid biosynthesis, alkaloid biosynthesis, plant hormone signal transduction, MAPK signaling pathway and metabolism of amino acids, glycerolipids and glycerophospholipids (**Figure 4E**). These pathways presumably play critical roles underlying resistance to tea green leafhopper infestation. By GO enrichment analysis, we identified 81 up-regulated and 17 down-regulated GO categories. More specifically, the up-regulated GO categories in LD mainly included genes involved in the biosynthesis of chitins, polysaccharides, lipids and fatty acids, protein modifications, and transport of organic acids (**Supplemental Table S9**). The down-regulated GO categories contained genes pertaining to amine metabolic process, tricarboxylic acid metabolic process and ion transport (**Supplemental Table S10**).

### Analysis of DEGs Reveals Important Genes Involved in Defensive Responses to Tea Green Leafhopper Infestation

We then performed a detailed analysis of DEGs potentially important for defensive responses to leaf herbivory in tea plants (**Table 2**). Plant hormone signaling network is usually activated after insect feeding, which in turn activate a cascade of downstream reactions. We found that genes involved in the biosynthesis and perception of plant hormones were notably affected by tea green leafhopper feeding. Auxin responsive GH3 gene family, auxin-responsive protein IAA, jasmonate ZIM domain-containing protein, cyclin D3, two-component response regulator ARR-A family, and DELLA protein were up-regulated in both “LD vs CK” and “LD vs MD” comparisons.

**Table 2:** List of significantly up-regulated and defense-related transcripts in tea green leafhopper-infested tea leaves.

Gene ID	Gene annotation	KO number	Fold change	
			LD vs CK	LD vs MD
**Phenylpropanoid biosynthesis**				
TEA024587	phenylalanine ammonia-lyase (PAL)	K10775	3.15	2.28
TEA023243	phenylalanine ammonia-lyase (PAL)	K10775	1.75	1.17
TEA014056	phenylalanine ammonia-lyase (PAL)	K10775	1.50	1.37
TEA014166	phenylalanine ammonia-lyase (PAL)	K10775	1.50	1.21
TEA034012	4-coumarate-CoA ligase (4CL)	K01904	1.64	1.37
TEA024897	cinnamyl alcohol dehydrogenase	K00083	2.90	2.11
TEA032966	cinnamyl alcohol dehydrogenase	K00083	1.54	1.41
TEA028587	*β*-glucosidase	K01188	2.71	1.39
TEA012891	peroxidase	K00430	2.60	1.13
TEA000197	peroxidase	K00430	1.72	1.11
TEA032135	shikimate *O*-hydroxycinnamoyltransferase	K13065	2.05	1.57
TEA030958	caffeoyl-CoA *O*-methyltransferase	K00588	1.64	1.28
**Flavonoid biosynthesis**				
TEA034003	chalcone isomerase (CHI)	K01859	1.76	1.22
TEA006847	flavonoid 3'-hydroxylase (F3'H)	K05280	2.39	1.28
TEA023790	flavanone 3-hydroxylase (F3H)	K00475	1.94	1.36
TEA006643	flavonol synthase (FLS)	K05278	1.79	1.72
TEA010328	flavonol synthase (FLS)	K05278	5.03	2.95
TEA032730	dihydroflavonol 4-reductase (DFR)	K13082	1.80	1.23
TEA027582	leucoanthocyanidin reductase (LAR)	K13081	1.58	1.61
**Plant-pathogen interaction**				
TEA026040	mitogen-activated protein kinase (MAPK)	K20536	7.80	6.57
TEA007881	calcium-binding protein CML	K13448	7.93	6.07
TEA016507	calcium-binding protein CML	K13448	5.39	2.90
TEA018321	calcium-binding protein CML	K13448	1.36	1.78
TEA003470	calcium-binding protein CML	K13448	1.76	1.54
TEA006621	calcium-dependent protein kinase	K13412	2.27	2.16
TEA027737	calcium-dependent protein kinase	K13412	1.86	1.63
TEA019257	calcium-dependent protein kinase	K13412	1.46	1.61
TEA001306	calmodulin	K02183	2.42	2.11
TEA005286	disease resistance protein RPM1	K13457	2.82	3.34
TEA002467	transcription factor WRKY	K13424	5.52	5.51
TEA007153	RPM1-interacting protein 4	K13456	1.60	2.66
TEA023870	chitin elicitor receptor kinase 1	K13429	2.61	3.03
TEA024230	enhanced disease susceptibility 1 protein	K18875	2.30	2.00
TEA011880	respiratory bust oxidase	K13447	2.81	1.80
TEA021188	serine/threonine-protein kinase PBS1	K13430	1.60	1.50
**Cutin, suberine and wax biosynthesis**				
TEA008365	aldehyde decarbonylase	K15404	22.31	41.20
TEA008367	aldehyde decarbonylase	K15404	3.64	9.74
TEA020004	fatty acid omega-hydroxylase	K15398	5.38	4.80
TEA015646	fatty acid omega-hydroxylase	K15398	1.90	4.66
TEA015695	omega-hydroxypalmitate *O*-feruloyl transferase	K15400	4.51	3.85
**Plant hormone signal transduction**				
TEA013731	auxin responsive GH3 gene family	K14487	1.83	10.91
TEA027708	auxin-responsive protein IAA	K14484	1.22	1.45
TEA032228	jasmonate ZIM domain-containing protein	K13464	7.91	6.24
TEA030190	jasmonate ZIM domain-containing protein	K13464	5.03	4.31
TEA014197	cyclin D3	K14505	2.28	2.06
TEA024232	two-component response regulator ARR-A family	K14492	1.35	1.60
TEA020933	DELLA protein	K14494	2.23	1.58

LD, tea green leafhopper-infested leaves; MD, mechanically damaged leaves; CK, undamaged control.

Among the up-regulated genes in LD, the most populated group was related to plant-pathogen interaction. Ca^2+^ ion plays an important role in mediating the cellular responses to biotic or abiotic stimuli (Ranty et al., 2016). As an integral component of stress signaling network, expression of genes encoding common calcium sensor proteins like calcium-binding protein, calcium-dependent protein kinase, and calmodulin showed significant induction in the current study, acting positively on herbivore resistance or defense against wounding (**Table 2**). Other relevant proteins in this category included MAPK, disease resistance protein RPM1, RPM1-interacting protein 4, chitin elicitor receptor kinase 1, enhanced disease susceptibility 1 protein, respiratory bust oxidase, and serine/threonine-protein kinase PBS1.

In addition, the LD group exhibited higher up-regulation of genes in cutin, suberine, and wax biosynthetic pathway such as aldehyde decarbonylase, fatty acid omega-hydroxylase and omega-hydroxypalmitate *O*-feruloyl transferase (**Table 2**). Of particular interest, we observed the most drastic change at more than 20 folds for transcript TEA008365 encoding aldehyde decarbonylase, a *CER1* homolog and the result was verified by qRT-PCR (**Supplemental Figure S3** in **Data Sheet 1**). This enzyme catalyzes decarbonylation of fatty aldehydes to produce long-chain hydrocarbons contributing to the formation of the cuticular wax, an active component of plant adaptations to biotic and abiotic stresses. The chemical compositions of the wax were implicated to be associated with plant-insect and plant-pathogen interactions (Bernard and Joubès, 2013). Hence, the substantial induction of this transcript after insect attack may suggest of its essential role in defense against herbivorous insects.

Finally, in the catechin biosynthetic pathway, the expression of genes encoding key metabolic enzymes such as phenylalanine ammonia-lyase (PAL), 4-coumarate-CoA ligase (4CL), chalcone isomerase (CHI), flavonoid 3′-hydroxylase (F3′H), flavanone 3-hydroxylase (F3H), flavonol synthase (FLS), dihydroflavonol 4-reductase (DFR), and leucoanthocyanidin reductase (LAR) all dramatically increased in LD, in line with the KEGG enrichment analysis. Pearson correlation analysis revealed high correlation coefficients between the transcript levels of the above genes and the contents of major flavanols and several other flavonoid compounds (**Supplemental Table S11**). For example, the expression pattern of *PAL* (TEA014166) significantly (*p* < 0.05) correlated with the contents of ECG, EC-ECG dimer, EGC-ECG dimer and kaempferol 3-*O*-glucosyl rutinoside. The expression pattern of *4CL* (TEA034012) showed statistically significant (*p* < 0.05) correlations with the contents of EGCG3“Me, ECG3”Me, EC, epiafzelechin 3-gallate and tricetin. Moreover, genes encoding cinnamyl alcohol dehydrogenase, peroxidase, shikimate *O*-hydroxycinnamoyltransferase, and caffeoyl-CoA *O*-methyltransferase in the phenylpropanoid pathway were also more induced in LD.

### Tea Green Leafhopper-Infested Tea Leaves Show Higher Accumulation of Very-Long-Chain Alkanes in Cuticular Waxes

**Figure 5:**
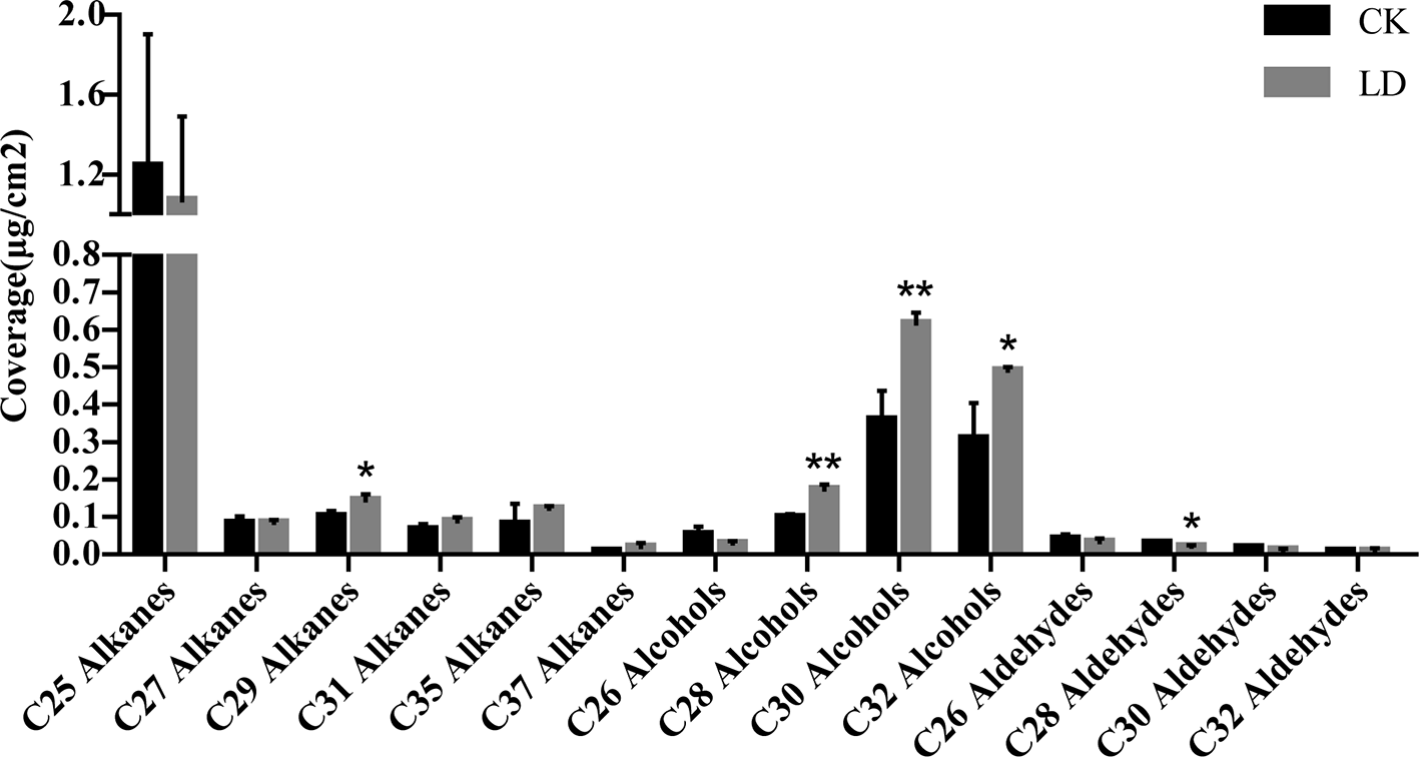
Analysis of abundant very-long-chain fatty acid derivatives in CK and LD leaf waxes. Error bars represent SD (n = 3). Statisical significance of differences between CK and LD means is indicated by *p < 0.05 and **p < 0.01. LD, tea green leafhopper-infested leaves; CK, undamaged control.

To further assess the effect of *CER1* induction on wax biosynthesis, cuticular wax compositions of leafhopper-damaged leaves (LD), and undamaged controls (CK) were analyzed *via* GC-MS and GC-FID. Representative chromatograms of tea samples and C_10_-C_40_ alkane standard mixtures were shown in **Supplemental Figure S4** in **Data Sheet 1**. In total, 354–393 compounds were detected from the wax mixtures of CK samples (n = 3) and 397–414 compounds were detected from those of LD samples (n = 3) (**Supplemental Table S12**). The wax mixtures on sampled tea leaves from both groups consisted mainly of fatty acids, alkanes, alkenes, aldehydes, and primary alcohols, similar to a recent report on the predominant wax constituents of tender tea leaves (Zhu et al., 2018) (**Table 3**). The wax load per unit of leaf area of LD leaves presented no drastic change compared to CK. Since *CER1* is mainly involved in very-long-chain (VLC) alkane biosynthesis, we specifically focused on the differences in VLC alkane levels between LD and CK leaf waxes. A further analysis of the chain length distribution within the alkane fractions indicated that they were characterized by odd-numbered homologs ranging from C25 to C37. C29 alkanes increased significantly (*p* < 0.05) in LD, showing a 43.6% increase compared to CK. Interestingly, there was also a large increase of primary alcohols (C28, C30, and C32) and a decrease in C28 aldehydes. Other alkanes and wax components were only weakly affected (**Figure 5**).

**Table 3:** Cuticular wax compositions of tea leaves from CK and LD groups.

Wax composition	CK (μg/cm^2^)	LD (μg/cm^2^)
Aldehydes	0.1 ± 0.01	0.07 ± 0.01
Alkanes	1.87 ± 0.71	1.79 ± 0.45
Alkenes	1.61 ± 0.27	1.66 ± 0.16
Esters	0.04 ± 0.01	0.04 ± 0.00
Ethers	0.08 ± 0.02	0.07 ± 0.01
Fatty acids	2.28 ± 0.38	1.82 ± 0.17
Primary alcohols	1.17 ± 0.29	1.79 ± 0.10
Total load	7.16 ± 1.12	7.25 ± 0.71

Values of the total wax loads and metabolite classes are shown as mean ± SD (n = 3).

### Major Transcription Factor Families Related to Stress Response Are Overrepresented in Tea Green Leafhopper-Infested Plants

Transcription factors (TFs) are master regulators that orchestrate the regulation of many different aspects of plant development and responses. Since transcriptional reprogramming is governed mostly by TFs, we therefore sought to identify differentially expressed TFs among different treatment groups. As illustrated in **Table 4**, comparisons of LD with CK identified 228 differentially expressed TFs, in which 153 were up-regulated and 75 were down-regulated. Comparisons of LD with MD identified 213 differentially expressed TFs, in which 138 were up-regulated and 75 were down-regulated. In contrary, only 31 TFs showed differential abundance with respect to mechanical damage. Major TF families such as AP2/ERF, bHLH, MYB, NAC, WRKY, and bZIP were documented to be closely related to defense signaling (Ng et al., 2018). In agreement with previous findings, we found that TF families with the most members overrepresented in LD were WRKY (24), AR2/ERF (24), bHLH (18), NAC (10) as well as GRAS (10). Potentially acting as a regulator of SA pathway, WRKY was the largest and the most significantly over-represented TF group, with all identified DEGs in this group being exclusively up-regulated (**Table 4**). Equally overrepresented was the AP2/ERF family, a regulator for the ERF-branch of the JA pathway. There were 24 up-regulated and 10 down-regulated genes in this family. Belonging to the bHLH family, MYC2 TF acts a master regulator of JA signaling pathway. Four MYC2-encoding transcripts, namely TEA009193, TEA012449, TEA016380, and TEA019380, were found to respond more strongly to the insect feeding than to the mechanical damage. Similarly, *NAC90*, *NAC2*, *SCL21*, and *SCL33*, members of NAC and GRAS TF families, were substantially up-regulated to a higher level in LD. These TFs could serve as candidates to potentially enhance resistance to tea green leafhoppers.

**Table 4:** List of differentially expressed transcription factors (TFs) identified by pairwise comparisons.

**TF family**	**LD/CK**		**LD/MD**		**MD/CK**	
	**up**	**down**	**up**	**down**	**up**	**down**
WRKY	24	0	23	0	0	0
AP2/ERF	24	10	25	9	2	4
bHLH	18	2	16	4	1	0
NAC	10	4	7	4	2	0
GRAS	10	1	8	1	1	1
MYB	8	8	10	6	2	0
bZIP	6	3	1	3	2	1
Tify	5	0	5	0	0	0
C2C2-GATA	4	0	3	0	0	0
C2C2-Dof	4	1	3	3	0	0
Orphans	3	1	1	2	0	0
OFP	3	0	0	0	1	0
HB	3	8	1	6	1	0
AUX/IAA	3	0	2	1	0	0
zf-HD	2	1	0	3	1	0
Trihelix	2	0	2	1	0	0
TCP	2	2	2	3	0	0
SNF2	2	0	1	0	0	0
SBP	2	2	3	3	0	0
LOB	2	3	2	2	0	2
HSF	2	2	2	1	0	0
GNAT	2	0	2	0	0	0
C2C2-YABBY	2	1	0	1	0	0
TAZ	1	0	1	0	0	0
RWP-RK	1	1	2	0	0	0
Pseudo ARR-B	1	0	1	0	0	0
G2-like	1	4	1	3	2	1
E2F-DP	1	0	1	0	0	0
DBP	1	0	1	0	1	0
CCAAT	1	1	4	0	0	0
C2H2	1	3	3	3	1	1
ARR-B	1	0	1	0	0	0
ARF	1	0	0	0	0	0
SWI/SNF-BAF60b	0	0	1	0	0	0
SRS	0	1	0	2	1	0
SET	0	1	1	0	0	0
PLATZ	0	1	0	1	0	0
mTERF	0	2	0	2	0	0
MADS	0	0	0	2	1	0
Jumonji	0	0	0	1	0	0
GRF	0	2	0	2	0	0
FHA	0	1	0	1	0	0
CPP	0	1	1	1	0	1
C3H	0	5	1	3	0	0
C2C2-CO-like	0	2	0	1	0	1
ABI3VP1	0	1	0	0	0	0
**sum**	153	75	138	75	19	12

LD, tea green leafhopper-infested leaves; MD, mechanically damaged leaves; CK, undamaged control.

## Discussion

Being a predominant and economically important piercing-sucking herbivore infesting tea plants, tea green leafhopper causes an average of 15%–50% annual loss in tea yield and a drastic decrease in tea quality in East Asia (Qin et al., 2015). Both nymphs and adults can suck phloem sap from tender shoots, buds, and young leaves, resulting in chlorosis, wrinkled leaves and premature leaf drop; in extreme cases, severely damaged tea plants would cease growing and ultimately die (Jin et al., 2012). To dissect the interaction between the tea plant and the tea green leafhopper, in the current study we characterized the comprehensive response of tea cultivar “Jinxuan” during its infestation by *E*. (*M*.) *onukii*. A global comparison of metabolome and transcriptome profiles was performed, complemented with the analyses of changes in leaf wax compositions and plant phytohormones. The results are discussed with special emphasis on plant defense responsive genes and metabolites induced by insect herbivory and the potential influence of hormone signaling crosstalk on eliciting insect response.

### Genes and Metabolites Involved in Phenylpropanoid/Flavonoid Biosynthesis Are Up-Regulated After Tea Green Leafhopper Infestation

Our results showed that tea green leafhopper infestation clearly triggered the direct defense responses in tea plants, including induced gene expression in the phenylpropanoid and flavonoid pathways, enhanced production of flavonoids, reinforcement of wax biosynthesis and accumulation of defense-related proteins. Phenylalanine metabolism, phenylpropanoid biosynthesis and flavonoid biosynthesis were among the most enriched pathways in tea plants following herbivore attack (**Figure 4**). More specifically, the insect feeding elevated the expression of genes encoding key metabolic enzymes PAL, 4CL, CHI, F3′H, F3H, FLS, DFR, and LAR pertaining to phenylpropanoid/flavonoid biosynthesis (**Table 2**). This was consistent with an early study showing the accumulation of transcripts for CHS (chalcone synthase), FLS and LAR in leafhopper-infested tea leaves (Yang et al., 2011). A similar induction of key genes in the phenylpropanoid pathway was observed when tea plants were damaged by tea geometrid (*Ectropis oblique*), a chewing insect (Wang et al., 2016). Therefore, it appears that the general up-regulation of the phenylpropanoid and flavonoid pathways is a common strategy employed by tea plants facing insect attacks regardless the feeding modes.

Correlated with gene expression analysis, some characteristic flavonoid compounds such as catechins, proanthocyanidin dimers, flavonol glycosides, flavone glycosides, and hydrolysable tannins drastically increased in their abundance in leafhopper-infested tea leaves (**Figure 2**), which further verifies that flavonoid compounds play a pivotal role in defense against tea green leafhopper feeding. In contrast, in a very recent study, the individual catechin and the total catechin contents in dissected tea leaves were not significantly altered by tea green leafhopper attack (Liao et al., 2019). Although in that study the infestation level by *E*. (*M*.) *onukii* was not mentioned, we speculate that the different results observed in two studies may result from different lengths of insect treatment (3 weeks in the current study *vs.* 48 h or 96 h in that study). Prolonged insect feeding might trigger more intense changes in defense gene expression, thus leading to more defense compound accumulation. Flavonoids, owing to their remarkable structural and functional diversity, fulfill a vast array of important functions in plant-insect interactions. Depending on the dosage applied and the insect species, this group of metabolites can provoke negative or stimulating effects on the feeding behaviors, survival and development of insects (Arimura et al., 2011). The defensive roles of flavonoids in plant protection against insect pests and herbivores occurs *via* altering plant palatability, reducing nutritive value, or act as feeding detergents or even toxins (Mierziak et al., 2014). For instance, rutin when applied at concentrations between 10^-4^ and 10^-5^ M stimulated the feeding of several species of *Spodoptera* and *Helicoverpa* but deterred the feeding of the same insects at higher concentrations (Schweiger et al., 2014). Likewise, supplemented at higher concentrations, flavonoids isorhamnetin 3-sophoroside-7-glucoside, and kaempferol 3,7-diglucoside acted as effective feeding deterrents against *Mamestra configurata* (Walk.) (Zust and Agrawal, 2017). In response to feeding by *Helicoverpa armigera* (a chewing insect) and *Aphis craccivora* (a sucking insect), peanuts (*Arachis hypogaea* L.) induced the accumulation of different flavonoid compounds (Yorozuya, 2017). The differentially accumulated flavonoid metabolites identified herein may serve as important biochemical markers for induced resistance against insect herbivores. Nevertheless, some questions remain to be answered regarding how the enhanced production of these molecules modulate resistance to leaf herbivory and whether they act additively or synergistically in feeding deterrence.

### Genes Involved in Cuticular Wax Alkane Biosynthesis Are Up-Regulated After Tea Green Leafhopper Infestation

Cuticle is a waterproof layer that covers the aerial parts of almost all terrestrial plants. It has been well known to act as a protective barrier against environmental stresses, protecting plants from water losses and UV radiation, defending against microbial pathogens, and participating in plant-insect interactions (Bernard and Joubès, 2013). Fortification of physical barriers like leaf cuticles and cell walls is an example of direct induced defenses.

In the current study, we observed the most drastic change in transcript abundance after leaf herbivory for transcript TEA008365, which increased by 21- and 40-fold compared with the undamaged control and mechanically damaged treatment. Its deduced amino acid sequence shares 62% sequence identity with CER1 protein, a VLC aldehyde decarbonylase catalyzing wax alkane formation in Arabidopsis (Baldwin and Preston, 1999). A second transcript TEA008367 induced by 3- and 9-fold compared with respective controls also encodes the same enzyme. As part of the cuticle, waxes comprise of a mixture of mostly very-long-chain fatty acids (VLCFAs) derivatives and variable amounts of triterpenoids, sterols and flavonoids, of which VLC alkanes are the major components formed through the alkane forming pathway (Jetter and Kunst, 2008; Kunst and Samuels, 2009). By analyzing *CER1*-overexpressing lines and TDNA insertional mutants, Bourdenx et al. has demonstrated that in Arabidopsis, *CER1* is the core element for VLC alkane biosynthesis and that modifying *CER1* expression alters wax composition and hence the cuticle permeability; more importantly, it is highly linked to plant responses to water deficit and pathogen attack (Bourdenx et al., 2011). Homologs of *CER1* have subsequently been identified and functionally characterized in crop plants such as cucumber, rice, and wheat, all confirming their key roles in VLC alkane biosynthesis, plant development and stress tolerance (Wang et al., 2015; Ni et al., 2018; Li et al., 2019). Despite extensive efforts to address the alkane-forming pathway for wax biosynthesis in different plants, the biochemical details have not been fully elucidated, especially in woody plants. In tea plants, investigations on wax biosynthesis and its involvement in responding to biotic and abiotic stresses have not yet been undertaken. In fact, there have only been a couple of reports comparing the cuticle and wax compositions between different tea cultivars or between leaf tissues from different developmental stages (Tsubaki et al., 2013; Zhu et al., 2018). It is not clear whether *CER1* homologs in tea plants perform the same function as in other plants. According to a recent bioinformatics analysis of 193 *CER1* sequences from 56 land plants, Wang et al. concluded that CER1 proteins are highly conserved throughout evolution (Wang et al., 2019). Given this, it is tempting to speculate that the aforementioned two *CER1* homologs in tea plants would have the same function.

A preliminary analysis of wax components of tea leaves sampled in the current study indicated that the proportion of C29 alkanes substantially increased in LD compared to CK while other VLC alkane levels were not largely affected. This might suggest that the differentially expressed *CER1* homolog is involved in VLC alkane biosynthesis and perhaps is mainly involved in C29 alkane biosynthesis in tea plants. Further study on VLC alkane biosynthetic pathway in tea plants is warranted. It will be intriguing to determine whether overexpressing genes in this pathway can confer enhanced resistance to herbivorous insects in tea plants.

### SA and JA Play Central Roles in Regulating Plant Responses to Tea Green Leafhopper Infestation

The activation of complex phytohormone signaling networks is a universal strategy employed by plants to decode insect-induced upstream signaling events into downstream specific defense responses (Ye et al., 2017). SA, JA, and ET are major hormones modulating inducible defenses while their interactions with other plant hormones such as ET, ABA, auxin, BR, CK, and melatonin are also important for priming the plant immune response (Yang et al., 2015).

On tea green leafhopper-infested tea plants, SA level increased substantially while JA level increased slightly (**Figure 3**), which appears to suggest that SA and JA pathways were simultaneously induced. These variations in phytohormone levels are in accord with the study by Liao et al. showing rise in both SA and JA contents of tea leaves infested by tea green leafhoppers and tea geometrids (Liao et al., 2019). However, the induction level of JA by tea green leafhoppers in the current study was much less compared to that study, which we anticipate was due to a longer insect treatment time employed. Rapid and efficient induction of defense hormones immediately following the recognition of an invader is essential for plants to launch defense reactions. On the initial encounter with insects, plants usually experience a quick surge in defense hormone production, whose levels during persistent insect infestation are often observed to level off or drop (Yang et al., 2019). This was also evident in the study by Liao et al. where they showed that JA level first sharply increased at 48 h post tea green leafhopper feeding but dropped by ~30% when sampled at 96 h (Liao et al., 2019).

It is thought that JA primarily induces resistance against tissue-damaging insects, necrotrophic pathogens and wounding whereas SA primarily induces resistance against biotrophic pathogens and insects causing little damage (Bari and Jones, 2009). In plants, SA and JA defense pathways usually antagonize each other as an adaptive strategy facing different invaders and their reciprocal antagonism has been confirmed in 17 plant species, including 11 crop plants and six wild species (Thaler et al., 2012). For example, SA-JA crosstalk has been well exemplified in tomato, showing that exogenous SA application could strongly inhibit gene expression in the JA pathway as well as JA-induced synthesis of proteinase inhibitors (Doares et al., 1995). In wheat, as in dicotyledonous plants, JA treatment suppressed SA responsive marker genes and vice versa (Ding et al., 2016). However, some notable exceptions exist that conflict with the SA-JA antagonism, which instead indicate synergistic interactions between SA and JA signaling pathways (Yang et al., 2015). For instance, during plant colonization by several spider mites, simultaneous induction of SA and JA signaling pathways was observed (Kant et al., 2004; Rioja et al., 2017; Arena et al., 2018). Similarly, a rice microarray analysis conducted by Tamaoki et al. demonstrated that more than half of genes up-regulated by SA were also up-regulated by JA (Tamaoki et al., 2013). In a study on *Plantago lanceolata*, it was shown that JA and SA treatment alone significantly reduced the survival of both *Heliothis virescens* (a chewing insect) and *Myzus persicae* (a piercing-sucking insect), but their combined application attenuated the negative effects towards *H. virescens*, suggesting that these two pathways are indeed connected by both divergence and convergence points (Schweiger et al., 2014).

Among the up-regulated hormonal pathways in response to tea green leafhopper infestation, the SA-mediated pathway seems to play the most dominant role given that several homologs of *PAL* genes responsible for synthesizing SA from cinnamate were significantly up-regulated, the WRKY TFs which may be involved in SA signaling were exclusively over-represented and the SA level was markedly elevated. Evidence has shown that induction of SA response is mainly linked to stealthy arthropods such as piercing-sucking insects (Arena et al., 2018). Likewise, the nymphs and adults of tea green leafhoppers pierce and ingest plant fluids from young tea shoots with their stylet mouthparts, causing little tissue damage. As a result, activation of SA pathway by tea green leafhoppers is consistent with the pattern commonly observed in piercing-sucking insects that lead to minimal tissue disruption (Arimura et al., 2011; Arena et al., 2018). In the current study, the continuous wounding by a microcapillary needle which is used to mimic the attack by a piercing-sucking insect did not elevate SA production in tea plants (**Figure 3**). This fact perhaps points to the existence of unknown elicitor(s) in tea green leafhoppers that cause differential phytohormone response in tea plants.

On the other hand, cases where the activation of SA pathway favoring herbivore performance rather than acting as an effective defense against herbivory have also been documented in various plants interacting with insects. For instance, oviposition by *Brevipalpus* mite on Arabidopsis mutants defective in SA biosynthesis and signaling was lower compared to that on wild-type Arabidopsis despite the fact that in the latter plant this insect infestation could simultaneously induce SA and JA production. Therefore, the authors view inducing the SA-mediated pathway as a strategy by this insect to manipulate the plant defensive responses to make the plant more susceptible to insect colonization (Arena et al., 2018). Likewise, when comparing the metabolic responses to tea geometrid attack between a susceptible tea cultivar and a resistant tea cultivar, researchers found that JA was more induced in the resistant cultivar whereas SA remained constantly in a higher level in the susceptible cultivar (Wang et al., 2018). All in all, these findings suggest that the interplay between SA and JA signaling pathways is highly complex, acting either antagonistically or synergistically to influence defense responses in different plant species. Given that different ecotypes of tea cultivars may react differently to the same insect species, a comparative approach using both resistant and susceptible tea cultivars to further explore the functional relevance of SA-JA crosstalk for resistant mechanisms towards tea green leafhopper attack is highly necessary.

### Reduction in Glutathione and Amino Acid Contents Is Likely the Result of Growth-Defense Tradeoffs Against Insect Herbivory

Defensive traits have been thought to be acquired by plants at the expense of other plant functions such as growth and reproduction (Zust and Agrawal, 2017). When attacked by insects or pathogens, the plant metabolism is usually reprogrammed towards enhancing specialized metabolism to ward off invaders while in the meantime the primary metabolism is often suppressed. For example, *Brevipalpus* mite infestation up-regulated defensive responses but down-regulated growth-related processes including those associated with photosynthesis, cell division, and morphogenesis of cell components (Arena et al., 2018). Similarly, we found that tea green leafhopper feeding down-regulated the processes involved in photosynthesis, cellular homeostasis, RNA polyadenylation, amine metabolism, and so on, providing evidence of trade-offs between defense and growth. In addition, the glutathione content markedly decreased and the total amino acid contents (mainly theanine and glutamate) in insect-infested tea leaves reduced by 59%. Again, this was in contrast with the study by Liao et al. where they saw no significant changes in the theanine contents in leafhopper-infested tea leaves (Liao et al., 2019). The discrepancies might arise from the duration of insect treatment time applied in two studies.

It is well established that plant amino N status greatly impacts insect N metabolism since sucking insects derive their diets from phloem sap, a tissue thought to be N-deficient. N nutrient availability in these phytophagous insects in turn influences their growth and reproduction (Crafts-Brandner, 2002; Guo and Ge, 2017). For instance, a study has shown that free amino acid pools and excretion of amino N in silverleaf whiteflies *Bemisia tabaci*, a major agricultural pest and a phloem feeder, could be rapidly altered by the N status of cotton leaves (Crafts-Brandner, 2002). Many other studies also suggest that increased nutrient availability positively benefits the performance of herbivorous insects with various feeding modes while negatively affecting plant specialized metabolism and thereby lowering plant resistance (Chow et al., 2009; Larbat et al., 2016; de Lange et al., 2019). Therefore, such a great reduction in amino acid and glutathione concentrations in tea leaves would presumably exert a large negative impact on insect performance and might deter further feeding. This might represent a strategy to enhance leaf resistance to tea green leafhopper infestation, a possibility that awaits further investigation. On the other hand, glutathione has been repeatedly reported to play essential roles in plant response to biotic and abiotic stresses (Zechmann, 2014). In particular, glutathione deficiency in a Arabidopsis mutant has been implicated to be associated with enhanced susceptibility to several fungal and bacterial pathogens (Dubreuil-Maurizi and Poinssot, 2012). It remains to be discovered whether the obvious reduction in the glutathione content after leaf herbivory would have a negative impact on tea plant stress tolerance.

Taken together, our results provide a comprehensive overview of the transcriptional, metabolic, and hormonal responses of tea plants to the tea green leafhopper, one of the most devastating pests affecting tea production in East Asia. We find that tea green leafhopper feeding triggers a drastic transcriptome and metabolome reprogramming in infested tea plants. Flavonoids accumulate to significant levels within tea leaves undergoing herbivore attack as a result of up-regulation of genes related to phenylpropanoid and flavonoid biosynthesis and they are the main contributors towards induced defense against insect herbivory. Substantial induction of genes encoding cuticular wax alkane biosynthesis upon the insect attack, which results in increased C29 alkane levels in tea leaf waxes, may play a key role in strengthening the resistance. Furthermore, tea green leafhopper feeding leads to increased production of two defense phytohormones SA and JA where SA-mediated pathway appears to play a more dominant role for the defense response. Future research employing multi-omics approaches to compare the responses between resistant and susceptible tea genotypes following the tea green leafhopper challenge will undoubtedly lead to a better understanding of resistance mechanisms.

## Data Availability Statement

The datasets generated for this study can be found in the NCBI Sequencing Read Archive database, accession number PRJNA553681.

## Author Contributions

XY and ZY conceived and designed the experiments. XZ, SC, SW, WS, XW, YL, and FS performed the experiments. XZ, SC, and XY analyzed the data. XZ, SC, ZY, and XY interpreted the results. XZ, ZY, and XY wrote the manuscript. All authors discussed the results and reviewed the final manuscript.

## Funding

This work was funded by the Distinguished Young Scholar Program of Fujian Agriculture and Forestry University (xjq201610) and the startup fund from Fujian Agriculture and Forestry University.

## Conflict of Interest

The authors declare that the research was conducted in the absence of any commercial or financial relationships that could be construed as a potential conflict of interest.
