# Supplementary material for: Defensive Responses of Tea Plants (Camellia sinensis) Against Tea Green Leafhopper Attack: A Multi-Omics Study
Source: Front Plant Sci. 2020 Jan 17;10:1705. doi: 10.3389/fpls.2019.01705 (PMC6978701; doi:10.3389/fpls.2019.01705)
Supplement: Supplementary file 1 [file DataSheet_1.pdf]

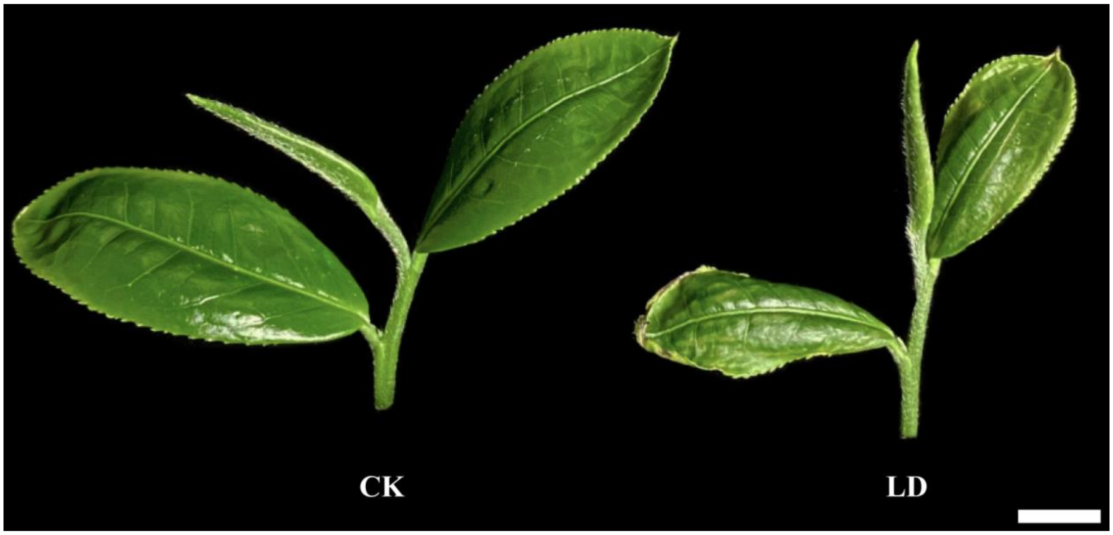

Supplemental Figure S1. Leaf phenotypes of green-leafhopper damaged (LD) tea leaves and undamaged tea leaves (CK). Bar, 1 cm.

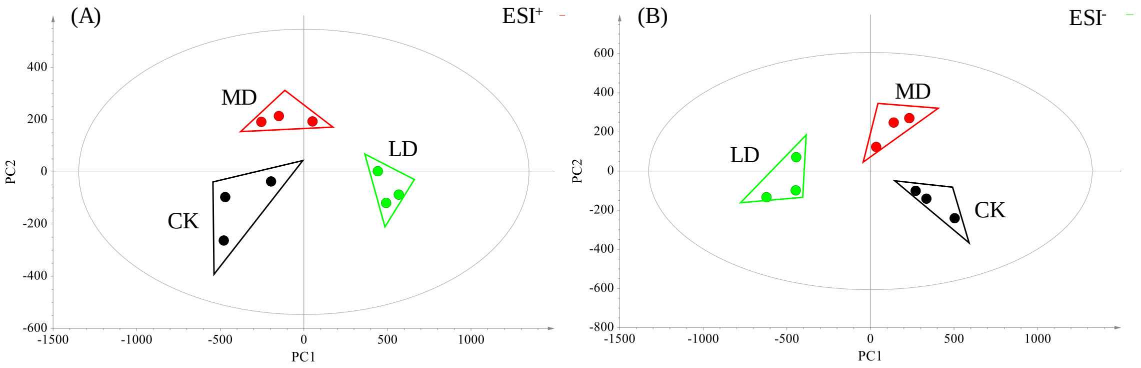

Supplemental Figure S2. PLS-DA analysis of tea leaves exposed to green leafhopper attack and mechanical damage. (A) PLS-DA score plot for tea samples based on 2,381 molecular features detected in ESI^{+}. (B) PLS-DA score plot for tea samples based on 906 molecular features detected in ESI^{-}. LD, tea green leafhopper-infested leaves. MD, mechanically damaged leaves. CK, undamaged control.

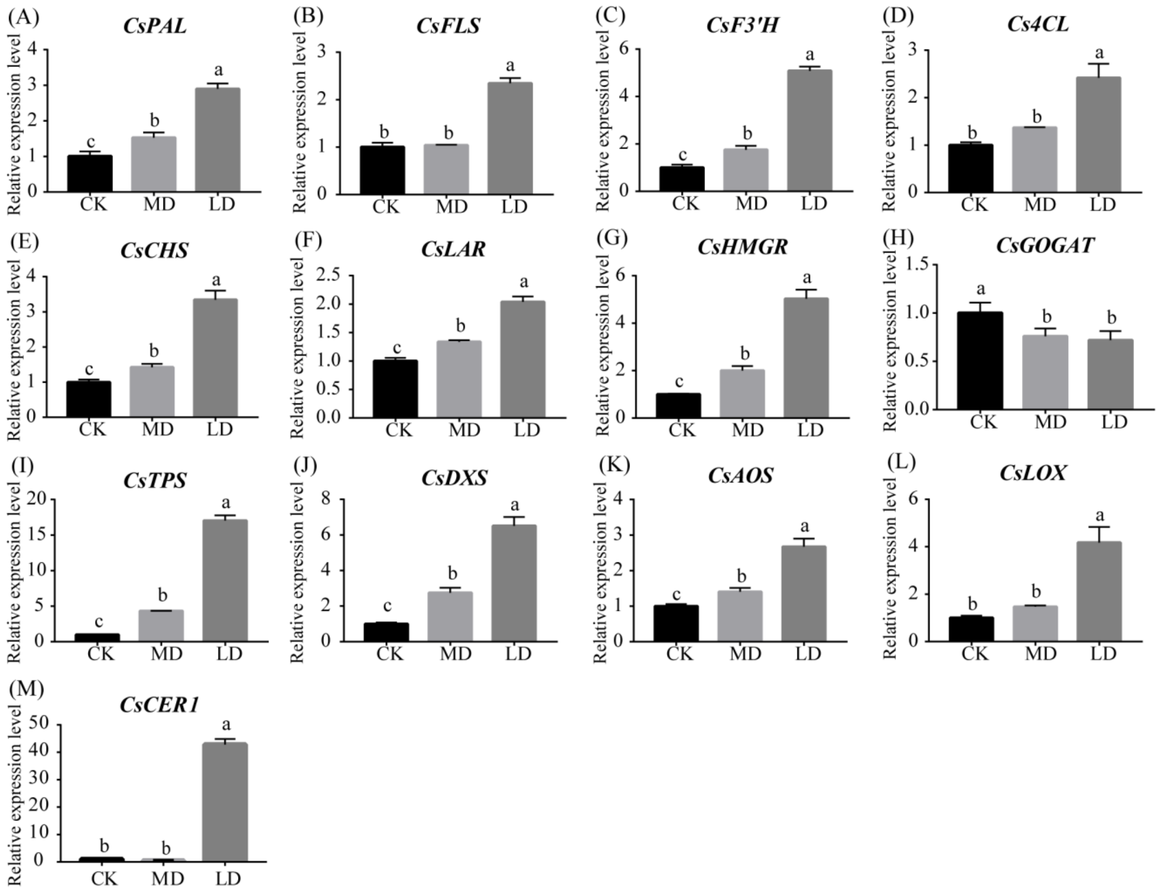

Supplemental Figure S3. Verification of the relative gene expression levels of selected genes by quantitative RT-PCR (qRT-PCR). (A) *PAL*, phenylalanine ammonia-lyase. (B) *FLS*, flavonol synthase. (C) *F3′H*, flavonoid 3′-hydroxylase. (D) *4CL*, 4-coumarate-CoA ligase. (E) *CHS*, chalcone synthase. (F) *LAR*, leucocyanidin reductase. (G) *HMGR*, 3-hydroxy-3-methyl-glutaryl-CoA reductase. (H) *GOGAT*, glutamate synthase. (I) *TPS*, terpene synthase. (J) *DXS*, 1-deoxy-D-xylulose 5-phosphate synthase. (K) *AOS*, allene oxide synthase. (L) *LOX*, lipoxydenase. (M) *CER1*, aldehyde decarbonylase. *GAPDH* (glyceraldehyde-3-phosphate dehydrogenase) gene was used as the internal control. Expression data are plotted as log2 values. The expression of genes in CK is set to 1.0. Different letters on top of the column indicate significant difference ($p<0.05$) according to Tukey’s HSD test. LD, tea green leafhopper-infested leaves. MD, mechanically damaged leaves. CK, undamaged control.

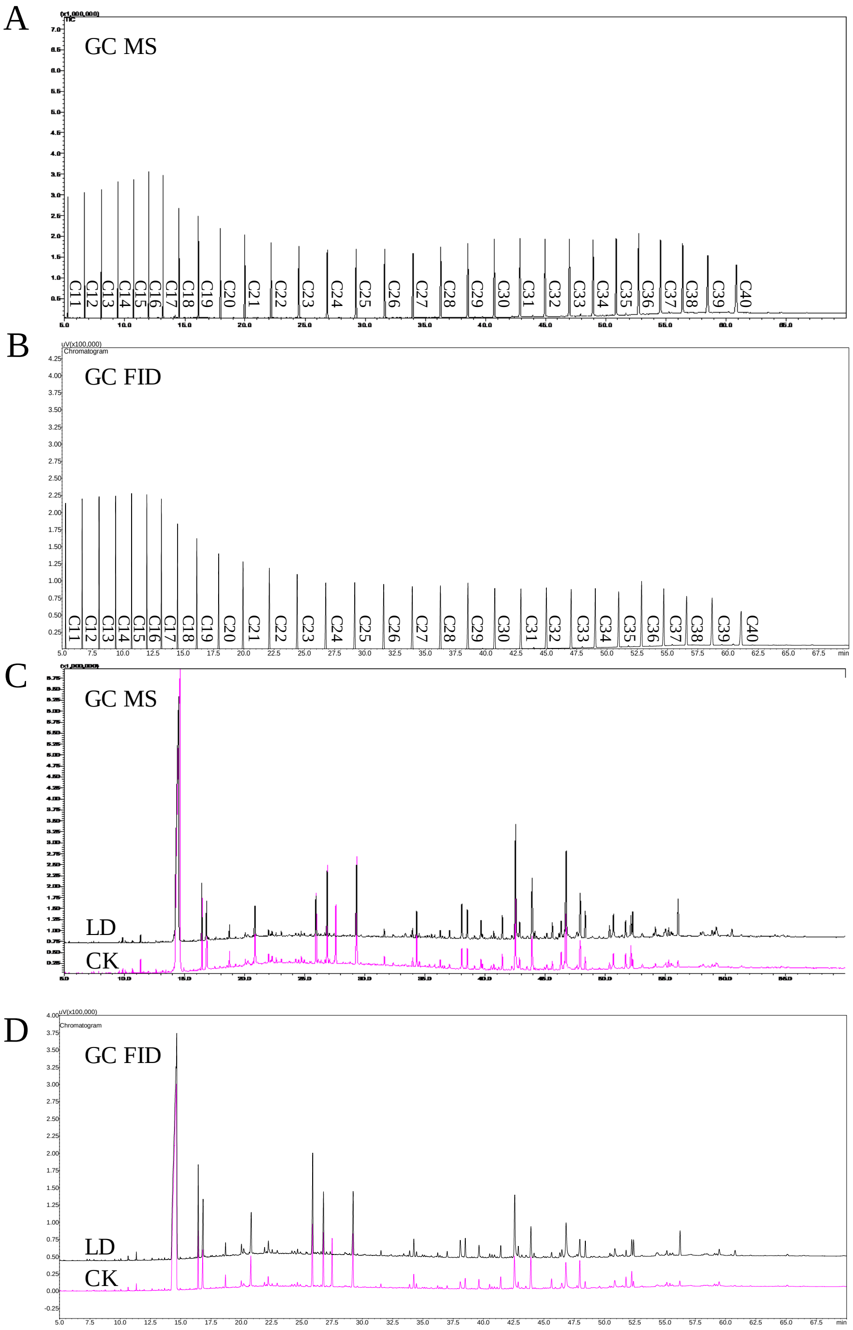

Supplemental Figure S4. Typical GC-MS (TIC) and GC-FID profiles for alkane standard mixtures (C10-C40) and representative samples. (A) GC-MS TIC chromatogram of mixed alkane standards. (B) GC-FID chromatogram profile of mixed alkane standards. (C) Overlaid GC-MS TIC chromatograms of cuticular wax components from CK and LD tea leaves. (D) Overlaid GC-FID chromatogram profiles of cuticular wax components from CK and LD tea leaves. LD, tea green leafhopper-infested leaves. CK, undamaged control.
