# Supplementary material for: Defensive Responses of Tea Plants (Camellia sinensis) Against Tea Green Leafhopper Attack: A Multi-Omics Study
Source: Front Plant Sci. 2020 Jan 17;10:1705. doi: 10.3389/fpls.2019.01705 (PMC6978701; doi:10.3389/fpls.2019.01705)
Supplement: Supplementary file 2 [file Table_1.docx]

**Supplemental Table S1**. Primers for qRT-PCR

| Primer name | Gene ID^a^ | Forward primer 5'-3′ | Reverse primer 5'-3′ |
| --- | --- | --- | --- |
| PAL (Phenylalanine ammonia-lyase) | TEA034008 | AGTTTCGGAAGCCGGTGGTG | CTGGCCTTCACTCCCTCCCT |
| 4CL (4-coumarate-CoA ligase) | TEA025906 | GGCGACACCTACACCTACGC | CGCCGAGGAAGGAGAACACG |
| CHS (Chalcone synthase) | TEA023333 | AGCCTCGTAGGCCAATCCCT | TTGGGCCGCTGAAACCAACT |
| FLS (Flavonol synthase) | TEA006643 | CCCGTGATCTCCCTCTCTCAAC | GGGCTCGACACCGTGATCTG |
| F3'H (Flavonoid 3′-hydroxylase) | TEA006847 | GCGGGTGATGTTGGGTCACA | GCGAGGACCATCAGCTCCAC |
| LAR (Leucocyanidin reductase) | TEA024761 | TGCTCCATGGTGATGTGTACGA | TGGCCGCAATGATCTTCACCT |
| LOX (Lipoxygenase) | TEA012289 | GCCCATGTCGGCTCCAATGA | ATTGCCTGTGTGCTGCCAGT |
| AOS (Allene oxide synthase) | TEA027984 | GTCGACGGTGTTCAGAGCCA | CCGGTGAAGACGTCTCGCTT |
| GOGAT (Glutamate synthase) | TEA011569 | GGTGGGACCGTTGTTGTGCT | TCGGGATTGGAACGTGGCAT |
| DXS (1-deoxy-D-xylulose 5-phosphate synthase) | TEA026768 | CGGTCCAGTCAATGGGCACAA | GGCGGATAGCCCTTGCCTTT |
| HMGR (3-hydroxy-3-methylglutaryl-coenzyme A reductase) | TEA002814 | TTGTTGCTCAACGCCTGCGA | CGCACCTCCACAAGCGTACA |
| TPS (Terpene synthase) | TEA004822 | ACGCCTAGGCATCGATTACCAT | GGCACATTGTACCCTTCTTGCCT |
| CER1 (Aldehyde decarbonylase) | TEA008365 | GAGAAGGGTTGAGAGAAGGAGA | GCCAATGAGGGTGTGTAGTGA |
| GAPDH (glyceraldehyde-3-phosphate dehydrogenase) | KA295375.1^b^ | TTGGCATCGTTGAGGGTCT | CAGTGGGAACACGGAAAGC |

^a^ Gene ID refers to the genome of *Camellia sinensis* var. Suchazao (http://tpia.teaplant.org/).

^b^ This refers to GenBank accession number.
