# Supplementary material for: Defensive Responses of Tea Plants (Camellia sinensis) Against Tea Green Leafhopper Attack: A Multi-Omics Study
Source: Front Plant Sci. 2020 Jan 17;10:1705. doi: 10.3389/fpls.2019.01705 (PMC6978701; doi:10.3389/fpls.2019.01705)
Supplement: Supplementary file 4 [file Table_3.docx]

**Supplemental Table S3**. Abundance (mg/g DW) of catechins, caffeine and amino acids in tea leaves based on UPLC-QqQ MS analysis

| Compound | CK | MD | LD |
| --- | --- | --- | --- |
| EGCG | 79.05±15.12 b | 100.99±13.78 ab | 118.25±15.86 a |
| EGC | 23.96±4.53 b | 30.12±2.68 b | 41.17±4.30 a |
| ECG | 4.08±0.81 c | 6.21±0.51 b | 8.70±0.84 a |
| EC | 1.63±0.46 b | 2.44±0.21 b | 3.68±0.63 a |
| EGCG3"Me | 1.20±0.65 b | 2.00±0.67 ab | 4.02±1.05 a |
| GC | 0.62±0.19 a | 0.55±0.13 a | 0.82±0.24 a |
| C | 0.02±0.01 a | 0.02±0.01 a | 0.04±0.01 a |
| Total catechins | 110.49±19.94 b | 142.40±17.04 ab | 176.69±22.53 a |
| Caffeine | 12.79±3.02 a | 16.86±3.30 a | 19.70±2.60 a |
| Theanine | 4.85±2.71 a | 5.12±1.34 a | 1.33±0.52 a |
| Glu | 3.52±0.50 a | 2.95±0.85 ab | 1.99±0.16 b |
| Asp | 1.21±0.06 a | 1.20±0.55 a | 0.72±0.05 a |
| Gln | 0.50±0.29 a | 0.22±0.03 a | 0.11±0.04 a |
| Ser | 0.28±0.15 a | 0.32±0.34 a | 0.12±0.02 a |
| Pro | 0.02±0.00 a | 0.01±0.00 a | 0.01±0.00 a |
| Val | 0.02±0.00 a | 0.02±0.01 a | 0.02±0.00 a |
| Leu | 0.01±0.00 a | 0.02±0.01 a | 0.01±0.00 a |
| IIe | 0.01±0.00 a | 0.01±0.01 a | 0.01±0.00 a |
| Lys | 0.04±0.01 a | 0.05±0.03 a | 0.03±0.00 a |
| Trp | 0.01±0.01 a | 0.01±0.01 a | 0.03±0.02 a |
| Phe | 0.02±0.00 a | 0.02±0.01 a | 0.01±0.00 a |
| Arg | 0.17±0.13 a | 0.28±0.32 a | 0.01±0.00 a |
| Tyr | 0.01±0.00 a | 0.01±0.01 a | 0.01±0.00 a |
| Met | ND | 0.01±0.00 a | ND |
| His | 0.01±0.01 a | 0.02±0.01 a | ND |
| GABA | 0.01±0.00 a | 0.01±0.00 a | ND |
| Total amino acids | 10.68±3.01 a | 9.64±3.00 ab | 4.40±0.80 b |

Results are expressed as mean ± standard deviation (n = 3). Means with different letters in row are significantly different according to Tukey’s HSD (honestly significant difference) test (*p* < 0.05). ND = non-detectable.
