# Supplementary material for: Defensive Responses of Tea Plants (Camellia sinensis) Against Tea Green Leafhopper Attack: A Multi-Omics Study
Source: Front Plant Sci. 2020 Jan 17;10:1705. doi: 10.3389/fpls.2019.01705 (PMC6978701; doi:10.3389/fpls.2019.01705)
Supplement: Supplementary file 5 [file Table_4.docx]

**Supplemental Table S4**. Sequencing and alignment statistics for RNA-seq datasets

| Samples | Clean bases | Clean reads | Q20 (%) | Q30 (%) | Mapped reads | Mapped (%) | Uniquely mapped (%) | Multimapped (%) |
| --- | --- | --- | --- | --- | --- | --- | --- | --- |
| CK_1 | 9.10G | 60665358 | 97.45 | 93.00 | 54465407 | 89.78 | 84.30 | 5.48 |
| CK_2 | 8.76G | 58430860 | 97.35 | 92.73 | 52150025 | 89.25 | 83.93 | 5.32 |
| CK_3 | 7.96G | 53099172 | 97.31 | 92.73 | 47556461 | 89.56 | 84.21 | 5.35 |
| MD_1 | 7.21G | 48072018 | 97.10 | 92.27 | 42777781 | 88.99 | 83.72 | 5.27 |
| MD_2 | 6.00G | 39972694 | 97.38 | 92.84 | 35581075 | 89.01 | 83.58 | 5.43 |
| MD_3 | 7.68G | 51171990 | 97.57 | 93.22 | 45916840 | 89.73 | 84.26 | 5.47 |
| LD_1 | 8.02G | 53440238 | 97.64 | 93.36 | 47773570 | 89.40 | 83.99 | 5.41 |
| LD_2 | 7.56G | 50375984 | 97.16 | 92.36 | 44925306 | 89.18 | 83.88 | 5.30 |
| LD_3 | 9.55G | 63638940 | 97.32 | 92.73 | 56687410 | 89.08 | 83.78 | 5.29 |
